# Evaluation of the Effects of Fucoidans from *Fucus* Species and *Laminaria hyperborea* against Oxidative Stress and Iron-Dependent Cell Death

**DOI:** 10.3390/md19100557

**Published:** 2021-09-29

**Authors:** Philipp Dörschmann, Sarah Apitz, Inga Hellige, Sandesh Neupane, Susanne Alban, Georg Kopplin, Signe Ptak, Xavier Fretté, Johann Roider, Marietta Zille, Alexa Klettner

**Affiliations:** 1 Department of Ophthalmology, University Medical Center, University of Kiel, Arnold-Heller-Str. 3, Haus 25, 24105 Kiel, Germany; Philipp.Doerschmann@uksh.de (P.D.); s-apitz@web.de (S.A.); Johann.Roider@uksh.de (J.R.); 2 Fraunhofer Research and Development Center for Marine and Cellular Biotechnology EMB, Mönkhofer Weg 239a, 23562 Lübeck, Germany; inga.hellige@t-online.de (I.H.); m.zille@uni-luebeck.de (M.Z.); 3 Institute for Medical and Marine Biotechnology, University of Lübeck, Mönkhofer Weg 239a, 23562 Lübeck, Germany; 4 Pharmaceutical Institute, Kiel University, Gutenbergstraße 76, 24118 Kiel, Germany; sneupane@pharmazie.uni-kiel.de (S.N.); salban@pharmazie.uni-kiel.de (S.A.); 5 Alginor ASA, Haraldsgata 162, 5525 Haugesund, Norway; Georg@alginor.no; 6 Department of Chemical Engineering, University of Southern Denmark, Campusvej 55, 5230 Odense, Denmark; sihp@igt.sdu.dk (S.P.); xafr@kbm.sdu.dk (X.F.); 7 Department of Pharmaceutical Sciences, Division of Pharmacology and Toxicology, University of Vienna, UZA II, Althanstraße 14, 1090 Vienna, Austria

**Keywords:** algae, ferroptosis, *F. vesiculosus*, *F. serratus*, *F. distichus* subsp. *evanescens*, hemorrhage, neurons, retinal pigment epithelium, uveal melanoma, seaweed

## Abstract

Fucoidans are algal polysaccharides that exhibit protective properties against oxidative stress. The aim of this study was to investigate different fucoidans from brown seaweeds for their ability to protect against iron-dependent oxidative stress (ferroptosis), a main hallmark of retinal and brain diseases, including hemorrhage. We investigated five new high-molecular weight fucoidan extracts from *Fucus vesiculosus*, *F. serratus*, and *F. distichus* subsp. *evanescens*, a previously published *Laminaria hyperborean* extract, and commercially available extracts from *F. vesiculosus* and *Undaria pinnatifida*. We induced oxidative stress by glutathione depletion (erastin) and H_2_O_2_ in four retinal and neuronal cell lines as well as primary cortical neurons. Only extracts from *F. serratus*, *F. distichus* subsp. *evanescens*, and *Laminaria hyperborea* were partially protective against erastin-induced cell death in ARPE-19 and OMM-1 cells, while none of the extracts showed beneficial effects in neuronal cells. Protective fucoidans also attenuated the decrease in protein levels of the antioxidant enzyme GPX4, a key regulator of ferroptosis. This comprehensive analysis demonstrates that the antioxidant abilities of fucoidans may be cell type-specific, besides depending on the algal species and extraction method. Future studies are needed to further characterize the health-benefiting effects of fucoidans and to determine the exact mechanism underlying their antioxidative abilities.

## 1. Introduction

Fucoidans, or sulfated fucans, are polysaccharides of algae cell walls containing fucose sugar and sulfate ester groups. Naturally, they are important for the integrity of the algae cell wall and fend off pathogens and other harsh environmental effects of the ocean and prevent dehydration [1,2]. They exhibit many different biological activities that depend on the origin of the extract, harvest time, the extraction process, chemical composition, structure, and size [3,4,5,6]. High-molecular weight fucoidans reduce inflammatory cell activation, cytokine release, and macrophage infiltration [7,8]. They are also antiangiogenic by counteracting vascular endothelial growth factor [4,9,10,11,12]. Furthermore, high-molecular weight fucoidans have been demonstrated to reduce oxidative stress [13,14].

Oxidative stress is the imbalance between the generation of reactive oxygen or lipid species and the ability of the cells to clear them. Oxidative stress is a hallmark of many retinal diseases, neurodegeneration, and brain injury [13,14]. It therefore presents an important therapeutic target. Importantly, iron has been implicated in oxidative stress [14]. While iron is essential for many proteins and cellular homeostasis, excessive iron can generate reactive oxygen and lipid species, reducing the antioxidant defense mechanisms of the cells and thereby leading to oxidative stress. Decades of research have demonstrated that excessive oxidative stress eventually results in cell death. In 2012, the term ferroptosis (from ferrous meaning “iron” and ptosis meaning “a fall”) was coined for a caspase-independent, iron-dependent form of regulated cell death activated by oxidative stress induced by reactive lipid species, glutathione depletion, and/or hemin (oxidized heme) [15,16,17].

Iron accumulation has been observed in a number of neurodegenerative diseases [14]. Furthermore, excessive iron is present after bleeding in the eye or in the brain, such as in subretinal, subarachnoid or intracerebral hemorrhage [18]. The cells that are mostly affected by oxidative stress in the eye are those of the retinal pigment epithelium (RPE) owing to their high lipid content, the phagocytic activity of the photoreceptor outer segment, and the abundance of mitochondria [13]. While RPE cells are crucial for oxidative stress protection in the retina, long-term exposure decreases their defense ability over time. In the brain, neurons are also significantly impacted by oxidative stress due to their limited energy storage compared to their high energy demand, as well as low antioxidant capacities and the high abundance of lipids in the brain [19,20]. Neurons depend on glutathione, which is an antioxidant itself but also a cofactor for glutathione peroxidase 4 (GPX4), an enzyme that reduces lipid peroxides into lipid alcohols [21]. Glutathione is a tripeptide requiring cystine import into the cells by the system X_c-_ transporter that exports glutamate while importing cystine. Neurons exposed to glutathione depletion or hemin have been demonstrated to die via ferroptosis [22,23,24,25].

The aim of this study was to evaluate the protective effects of different fucoidans against oxidative stress induced by glutathione depletion (erastin, a system X_c-_ inhibitor) and H_2_O_2_ in retinal and neuronal cells.

## 2. Results

### 2.1. Characterization of Fucoidans

We produced five new high-molecular weight fucoidan extracts, from *F. vesiculosus* (FV1, FV2, FV3, different extraction methods), *F. serratus* (FS), and *F. distichus* subsp. *evanescens* (FE), which we compared to our previously published extract from *Laminaria hyperborea* (Fuc1 [4]) and the commercially available Fucoidan extracts from Sigma-Aldrich (*F. vesiculosus*, FVs, [26]) and Marinova (*F. vesiculosus*, FVm (GRAS No. 661)) and *Undaria pinnatifida*, UPm (GRAS No. 565) (Table 1). The degree of sulfation ranged from 0.26 to 1.70, with Fuc1 containing the highest sulfate content and FV3 containing the lowest. In addition, FV2 and FE consisted of a considerable amount of protein.

Acetylation analysis revealed that fucose was the dominant monosaccharide (50.5–87.1%) in each sample. In addition, FV1 consisted of a considerable amount of glucose (34.2%), which may represent the co-extracted laminarin in the fucoidan sample [11], while FV2 consisted of a remarkable amount of xylose (19.8%). To note, we used different extraction conditions, which resulted in different monosaccharide compositions from the same alga (FV1, FV2 and FV3, Table 1).

The molecular mass (M) and size characteristics of the new fucoidan samples were analyzed by size-exclusion chromatography with triple detection, i.e., multi-angle light scattering (MALS), viscometry and refractive index (RI). MALS/RI provided the weight-average and number-average molecular weights (M_W_, M_n_) and the root-mean square radius (rg), and the viscometer allowed us to measure the hydrodynamic radius (rh) and intrinsic viscosity [ŋ] of the samples. The M_W_ ranged from 52–1548 kDa (Table 2). The different extraction methods used resulted in a large variation in M_W_ (FV1: 261 kDa and FV2: 1438 kDa) among fucoidans from the same alga (*F. vesiculosus*). The fucoidan from Sigma-Aldrich (FVs) was the smallest one (52 kDa), whereas Fuc1 was the largest (1548 kDa).

**Figure 1:**
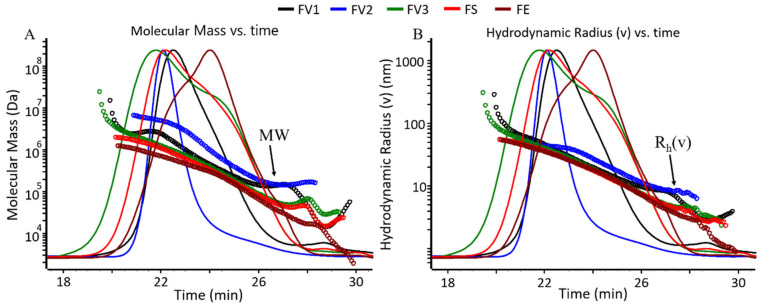
Characterization of the new fucoidan extracts. (**A**) Molecular mass-versus-elution time and (**B**) hydrodynamic radius-versus-elution time plots of the new fucoidan extracts from *F. vesiculosus* (FV1, FV2, FV3), *F. serratus* (FS) and *F. evanescens* (FE). LS (90° angle) chromatogram is overlayed in both plots.

According to the M_W_- and rh-versus-retention time plots, the fucoidans were heterogeneously composed and differed from each other (Figure 1A,B). Fuc1 was previously described in Dörschmann et al. (2020) as a large random coil shape molecule [4]. The M_W_ and rh of the samples in this study showed a very broad distribution. After a regular decrease, both M_W_ and rh rose slightly towards the higher elution time, which is quite common for branched as well as charged molecules [28].

**Figure 2:**
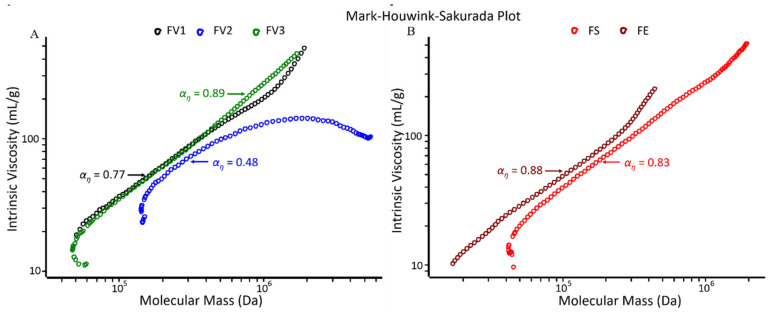
Mark–Houwink–Sakurada plots for (**A**) the three fucoidans from *F. vesiculosus* (FV1, FV2, FV3) and (**B**) the *F*. *serratus* and *F*. *evanescens* extracts (FS and FE, respectively).

In addition, the overall shape of the fucoidan samples was determined by Mark–Houwink–Sakurada (MHS) analysis, where [ŋ] is plotted against M_W_. The MHS slope (αŋ) provides information about the conformation of macromolecules, whereby a value of 0.0 corresponds to an ideal solid sphere, 0.5–0.8 to random coils, and 0.8–1.8 to rigid chains [29]. The MHS slope values of the main fraction of all fucoidans (except FV2) aligned with the theoretical value of random coil conformation (αŋ = 0.77–0.89, Figure 2A,B). Additionally, Fuc1 was described to have a random coil structured main chain with highly branched short side chains [4]. In contrast, the lower MHS slope (αŋ = 0.48) of FV2 indicated a more compact overall structure, i.e., a random coil tending towards a spherical shape.

To note, it can be observed in the Mark–Houwink–Sakurada plot that the slope of FV2 is much smaller than that of FV3 (Figure 2A), representing a lower intrinsic viscosity due to the different degree of branching present in these two fucoidans. In general, branched molecules have lower viscosity in comparison to linear molecules [30,31]. In addition, the slope bends downwards at the high-MW region, which is due to the lower viscosity of the high-MW molecules. FV3 represents a random coil structure while FV2 is a comparatively compact structure. This is the reason why the smaller Mw fucoidans in this study had a higher average intrinsic viscosity.

### 2.2. Protective Effect of Fucoidans against Erastin- and H_2_O_2_-Induced Cell Death

**Figure 3:**
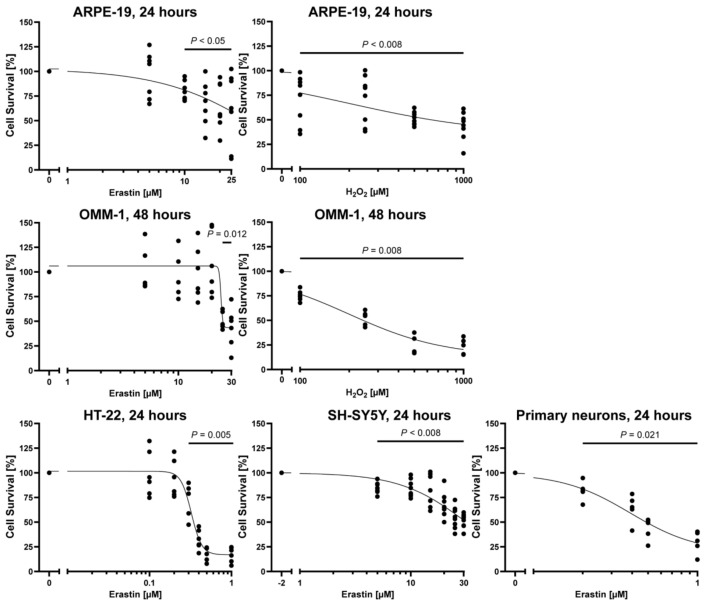
Erastin and H_2_O_2_ concentration responses. Cells were exposed to different concentrations of erastin or H_2_O_2_ (at logarithmic scale) for 24 or 48 h. Cell survival was normalized to vehicle controls, which were set to 100%. Concentration responses show biological replicates around the regression. *n* = 5–8. *p*-values are vs. vehicle treatment. For detailed statistics, please refer to Table S1.

We first determined the half-maximal lethal dose of erastin and H_2_O_2_ in the human RPE cell line ARPE-19, the uveal melanoma cell line OMM-1, the mouse hippocampal neuronal cell line HT-22, the human neuroblastoma cell line SH-SY5Y, and primary mouse cortical neurons (Figure 3). We used primary mouse cortical neurons because they more closely resemble neurons in vivo compared to cell lines, including their inability to proliferate, which may be of relevance to their resistance to oxidative stress and iron-dependent cell death.

While HT-22 cells and primary cortical neurons were very sensitive to erastin treatment at 24 h (*p* = 0.005 for 0.3–1 µM erastin vs. vehicle for HT-22 cells, *p* = 0.021 for 0.2–1 µM for primary cortical neurons), ARPE-19 cells only reached half-maximal lethal survival at 20 µM (*p* = 0.004), SH-SY5Y cells at 30 µM erastin (*p* = 0.002) after 24 h, and OMM-1 cells were only sensitive after 48 h with a half-maximal lethal dose at 25 µM (*p* = 0.012). For H_2_O_2_-induced death, ARPE-19 cells reached half-maximal lethality at 500 µM H_2_O_2_ after 24 h (*p* = 0.001) and OMM-1 at 250 µM H_2_O_2_ after 48 h (*p* = 0.008).

Next, we assessed the different fucoidan extracts at the half-maximal lethal dose in the different cell types. Fucoidan extracts were added 30 min prior to erastin or H_2_O_2_ treatment. For all cell lines, fucoidan concentrations between 0 and 50 μg/mL were applied. For primary cortical neurons, fucoidan concentrations of 0–5 μg/mL were used, because higher concentrations led to cell detachment due to the negative charge of fucoidans that interacted with the poly-l-lysine coating required for primary cortical neurons. Ferrostatin-1 was used as a positive control for erastin-induced oxidative stress in neurons.

**Figure 4:**
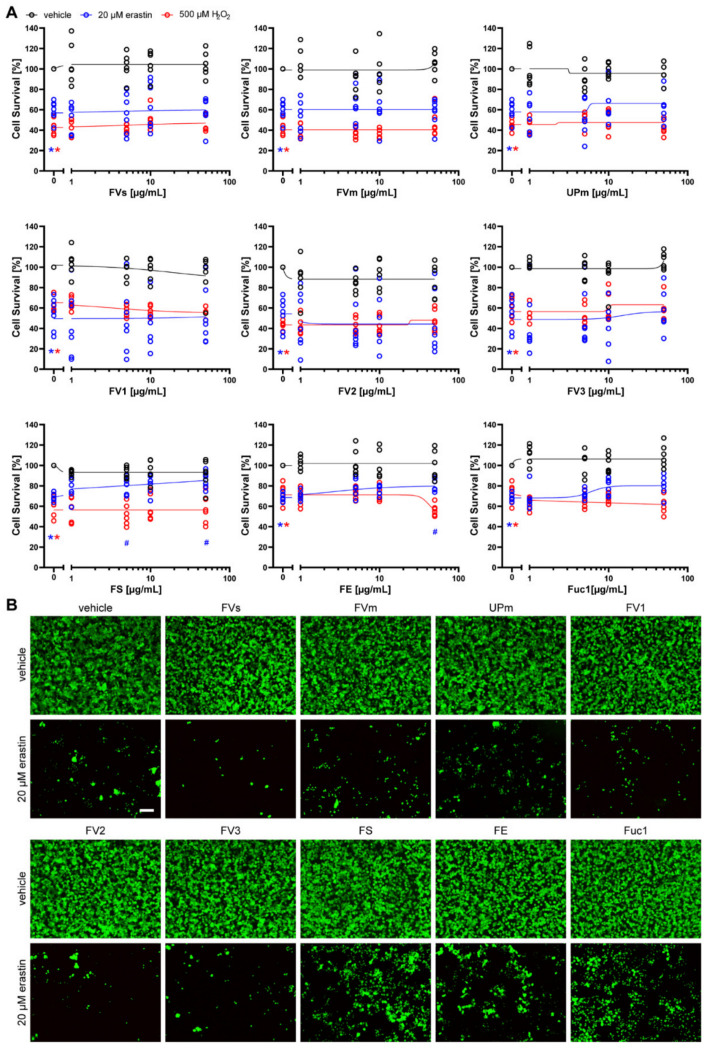
Fucoidans in erastin- and H_2_O_2_-induced death in ARPE-19 cells. (**A**) ARPE-19 cells were exposed to 1, 5, 10 or 50 µg/mL fucoidan (presented in logarithmic scale) 30 min prior to treatment with 20 µM erastin or 500 µM H_2_O_2_ for 24 h. Cell survival was normalized to vehicle controls, which were set to 100%. Concentration responses show biological replicates around the regression. Black lines indicate treatment of ARPE-19 cells with increasing fucoidan concentrations without erastin or H_2_O_2_ treatment. Blue lines indicate treatment of ARPE-19 cells with increasing fucoidan concentrations and 20 µM erastin. Red lines indicate treatment of ARPE-19 cells with increasing fucoidan concentrations and 500 µM H_2_O_2_. Medians are given. *n* = 6–7. * *p* < 0.05 compared to vehicle treatment without erastin or H_2_O_2_. # *p* < 0.05 compared to 20 µM erastin plus vehicle. For detailed statistics, please refer to Tables S2 and S3. (**B**) Representative calcein AM staining of 50 µg/mL fucoidan with and without 20 µM erastin is shown. Scale bar = 100 µm.

First, we investigated ARPE-19 cells (Figure 4). While cell death induced by erastin (median: 63.59%) was significantly abrogated by 50 µg/mL of the fucoidan extracts FS (median: 85.71%, *p* = 0.016) and FE (median: 80.47%, *p* = 0.026), 50 µg/mL Fuc1 treatment led to similar median protective levels, but was not significant due to high variability in the data (median: 81.15%, *p* = 0.164) (Figure 4A). These partial protective effects were confirmed using calcein AM staining (Figure 4B), which further showed that none of the fucoidans was toxic to the cells, as indicated by the MTT values without erastin treatment. In contrast, none of the extracts was protective against H_2_O_2_-induced cell death in ARPE-19 cells (Figure 4A).

**Figure 5:**
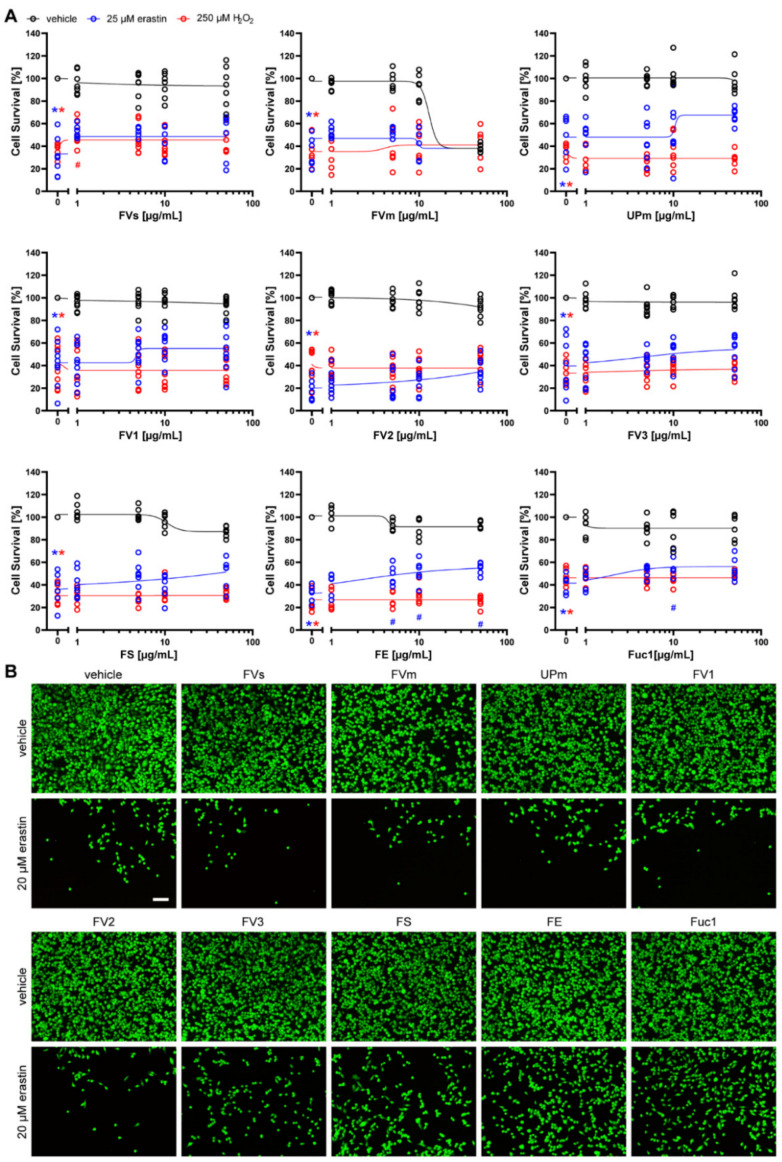
Fucoidans in erastin- and H_2_O_2_-induced death in OMM-1 cells. (**A**) OMM-1 cells were exposed to 1, 5, 10 or 50 µg/mL fucoidan (presented in logarithmic scale) 30 min prior to treatment with 25 µM erastin or 500 µM H_2_O_2_ for 24 h. Cell survival was normalized to vehicle controls, which were set to 100%. Concentration responses show biological replicates around the regression. Black lines indicate treatment of OMM-1 cells with increasing fucoidan concentrations without erastin or H_2_O_2_ treatment. Blue lines indicate treatment of OMM-1 cells with increasing fucoidan concentrations and 25 µM erastin. Red lines indicate treatment of OMM-1 cells with increasing fucoidan concentrations and 250 µM H_2_O_2_. Medians are given. *n* = 6–9. * *p* < 0.05 compared to vehicle treatment without erastin or H_2_O_2_. # *p* < 0.05 compared to 25 µM erastin or 500 µM H_2_O_2_ plus vehicle. For detailed statistics, please refer to Tables S4 and S5. (**B**) Representative calcein AM staining of 50 µg/mL fucoidan with and without 25 µM erastin is shown. Scale bar = 100 µm.

In OMM-1 cells (Figure 5A), FE showed a concentration-dependent significant protective effect (medians: 5 µg/mL 46.72%, *p* = 0.019; 10 µg/mL 52.12%, *p* = 0.021; 50 µg/mL 56.17%, *p* = 0.016) after erastin treatment (median: 34.73%). Furthermore, 10 µg/mL Fuc1 abrogated erastin toxicity (median: 59.48%, *p* = 0.026). Additionally, 50 µg/mL of FVs (median: 60.71%, *p* = 0.128), UPm (median: 70.22%, *p* = 0.072), FV3 (median: 58.06%, *p* = 0.992), and FS (median: 55.31%, *p* = 0.101) had higher MTT values; however, they were not significantly increased due to high variability in the data. Calcein AM staining revealed that the cell viability was partially increased by FS, FE and Fuc1 in the presence of erastin (Figure 5B). Furthermore, 50 µg/mL FVm led to a decrease in metabolic activity even without erastin or H_2_O_2_ treatment. This was at least partially due to cell death and detachment of the cells, which we observed in the calcein AM images. In contrast, only 1 µg/mL FVs led to a partial, but significant increase in cell viability (median: 48.55%, *p* = 0.035) in H_2_O_2_-induced cell death (median: 38.33) (Figure 5A). However, this single positive result may also be attributed to chance. Hence, our data suggest that, overall, the investigated fucoidans were not protective against H_2_O_2_-induced cell death in ARPE-19 or OMM-1 cells.

**Figure 6:**
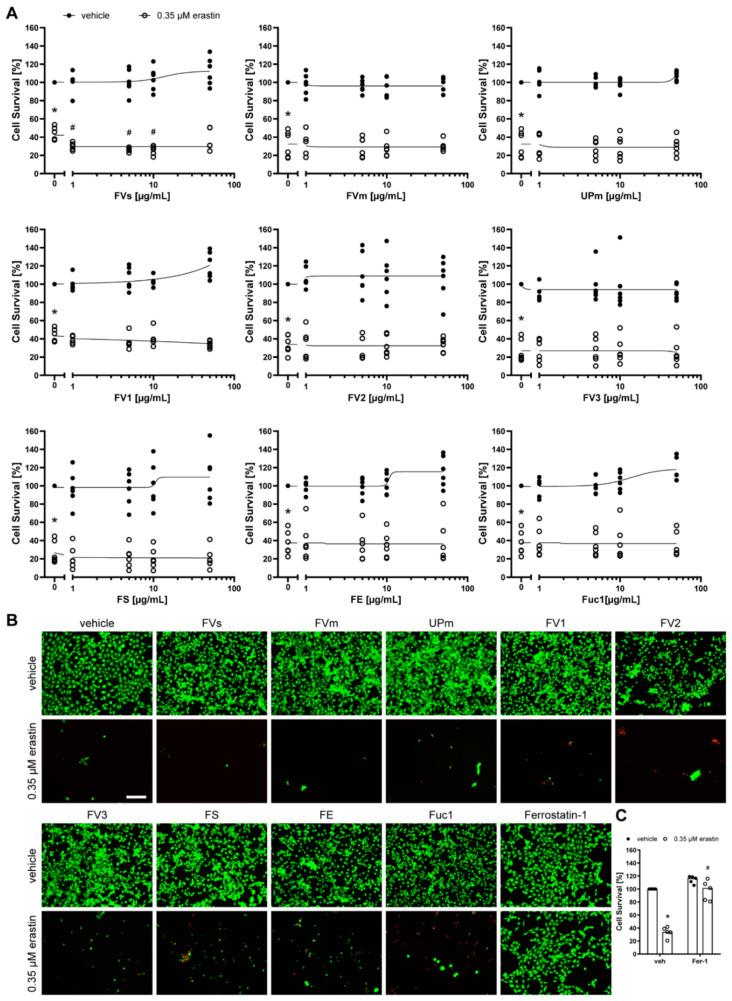
Fucoidans did not abrogate erastin-induced death in HT-22 cells. (**A**) HT-22 cells were exposed to 1, 5, 10 or 50 µg/mL fucoidan (presented in logarithmic scale) 30 min prior to treatment with 0.35 µM erastin for 24 h. Cell survival was normalized to vehicle controls, which were set to 100%. Concentration responses show biological replicates around the regression. Closed circles indicate treatment of HT-22 cells with increasing fucoidan concentrations without erastin treatment. Open circles indicate treatment of HT-22 cells with increasing fucoidan concentrations and 0.35 µM erastin. *n* = 6. (**B**) Representative live/dead stainings of 50 µg/mL fucoidan or 0.5 µM ferrostatin-1 with and without 0.35 µM erastin are shown, with green indicating live cells (calcein AM) and red indicating dead cells (propidium iodide). Scale bar = 100 µm. (**C**) HT-22 cells were exposed to vehicle or 0.5 µM ferrostatin-1 (Fer-1) 30 min prior to treatment with 0.35 µM erastin for 24 h. Medians are given. *n* = 5. * *p* < 0.05 compared to vehicle treatment without erastin. # *p* < 0.05 compared to 0.35 µM erastin plus vehicle. For detailed statistics, please refer to Table S6.

**Figure 7:**
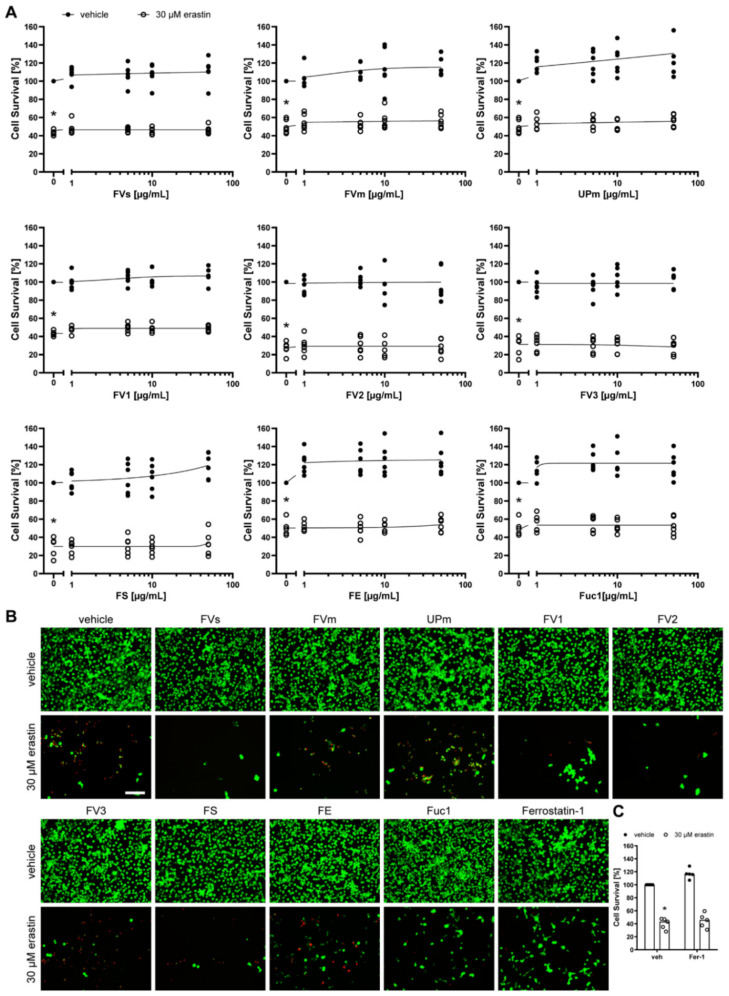
Fucoidans did not abrogate erastin-induced death in SH-SY5Y cells. (**A**) SH-SY5Y cells were exposed to 1, 5, 10 or 50 µg/mL fucoidan (presented in logarithmic scale) 30 min prior to treatment with 30 µM erastin for 24 h. Cell survival was normalized to vehicle controls, which were set to 100%. Concentration responses show biological replicates around the regression. Closed circles indicate treatment of SH-SY5Y cells with increasing fucoidan concentrations without erastin treatment. Open circles indicate treatment of SH-SY5Y cells with increasing fucoidan concentrations and 30 µM erastin. *n* = 6. (**B**) Representative live/dead stainings of 50 µg/mL fucoidan or 10 µM ferrostatin-1 with and without 30 µM erastin are shown, with green indicating live cells (calcein AM) and red indicating dead cells (propidium iodide). Scale bar = 100 µm. (**C**) SH-SY5Y cells were exposed to vehicle or 10 µM ferrostatin-1 (Fer-1) 30 min prior to treatment with 30 µM erastin for 24 h. Medians are given. *n* = 5. * *p* < 0.05 compared to vehicle treatment without erastin. For detailed statistics, please refer to Table S7.

**Figure 8:**
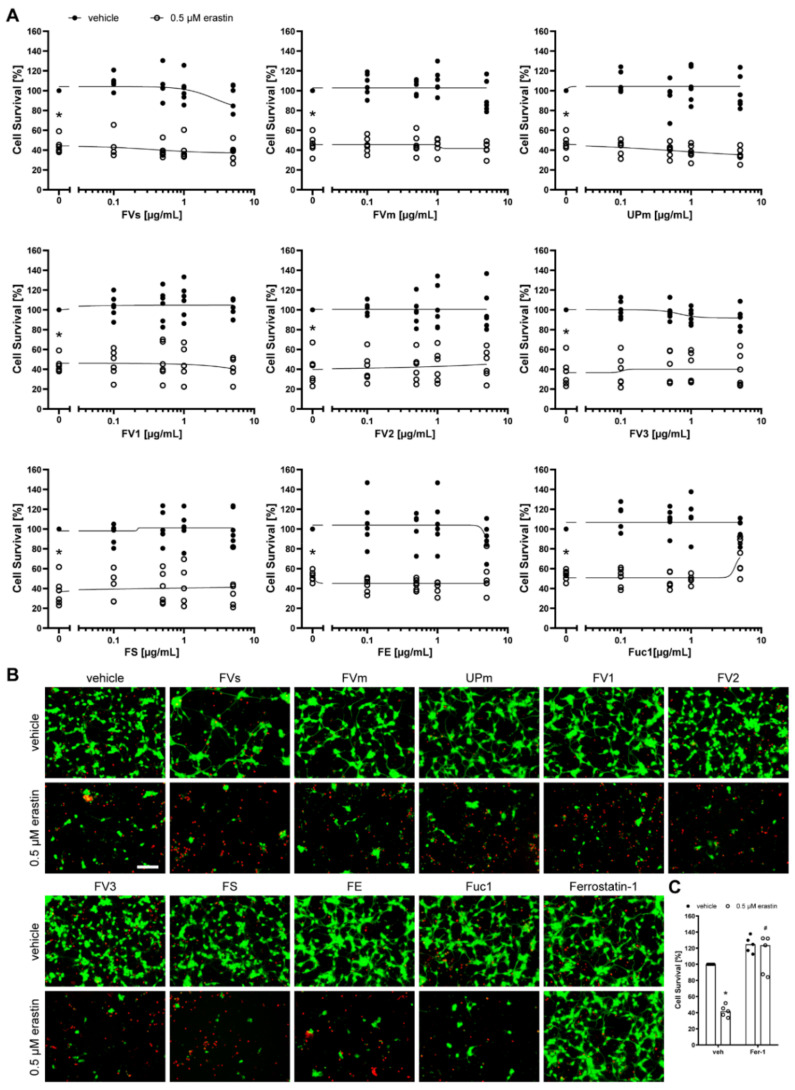
Fucoidans did not abrogate erastin-induced death in primary cortical neurons. (**A**) Primary cortical neurons were exposed to 0.1, 0.5, 1 or 5 µM fucoidan (presented in logarithmic scale) 30 min prior to treatment with 0.5 µM erastin for 24 h. Cell survival was normalized to vehicle controls, which were set to 100%. Concentration responses show biological replicates around the regression. Closed circles indicate treatment of primary cortical neurons with increasing fucoidan concentrations without erastin treatment. Open circles indicate treatment of primary cortical neurons with increasing fucoidan concentrations and 0.5 µM erastin. *n* = 6. (**B**) Representative live/dead stainings of 5 µM fucoidan or 0.5 µM ferrostatin-1 with and without 0.5 µM erastin are shown, with green indicating live cells (calcein AM) and red indicating dead cells (propidium iodide). Scale bar = 100 µm. (**C**) Primary cortical neurons were exposed to vehicle or 0.5 µM ferrostatin-1 (Fer-1) 30 min prior to treatment with 0.5 µM erastin for 24 h. Medians are given. *n* = 5. * *p* < 0.05 compared to vehicle treatment without erastin. # *p* < 0.05 compared to 0.5 µM erastin plus vehicle. For detailed statistics, please refer to Table S8.

Next, we assessed the neuronal cells HT-22 (Figure 6), SH-SY5Y (Figure 7), and primary cortical neurons (Figure 8) after treatment with the nine fucoidan extracts and erastin at the respective half-maximal lethal dose for 24 h. None of the extracts was protective against erastin-induced toxicity, which was also confirmed by calcein AM/propidium iodide staining. They also did not induce any toxicity when incubated without erastin. However, it has to be noted that primary cortical neurons were only treated with up to 5 µg/mL due to cell detachment at higher concentrations. In contrast, the positive control, ferrostatin-1, which prevents lipid peroxidation [32], was able to significantly increase survival of HT-22 cells (median: 101.43%, *p* = 0.011, Figure 6C) and primary cortical neurons (median: 123.59%, *p* = 0.011, Figure 8C), but not of SH-SY5Y cells (median: 45.74%, *p* = 0.465, Figure 7C). The latter inability may be explained by the much higher concentration of erastin needed to induce cell death in SH-SY5Y cells.

Taken together, the most promising fucoidan extracts were those from *F. serratus* (FS), *F. distichus* subsp. *evanescens* (FE), and *L. hyperborea* (Fuc1), which increased cell viability in erastin-induced cell death in ARPE-19 and OMM-1 cells. A summary of the effects of all the extracts at 50 µg/mL (5 µg/mL for primary cortical neurons) depending on the cell types is presented in Table 3.

### 2.3. Fucoidans FS, FE, and Fuc1 Abrogated the Decrease in GPX4 Protein Expression Induced by Erastin

**Figure 9:**
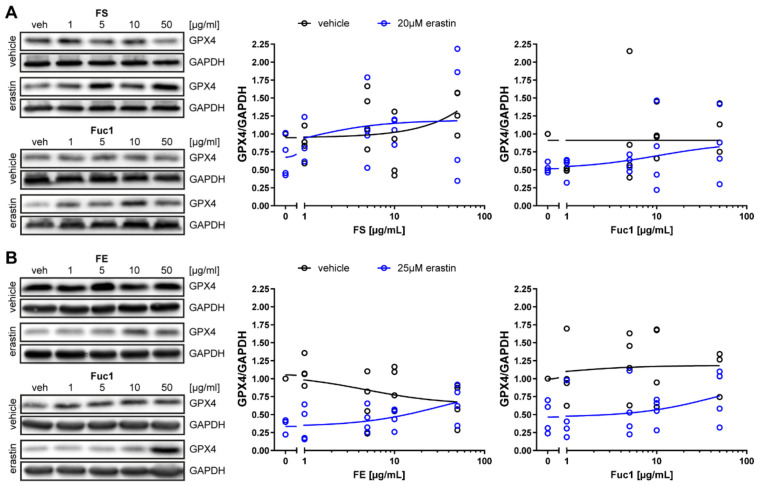
Fucoidans attenuated the decrease in GPX4 protein levels induced by erastin. (**A**) ARPE-19 cells were exposed to 1, 5, 10 or 50 µM fucoidan 30 min prior to treatment with 20 µM erastin for 24 h. (**B**) OMM-1 cells were exposed to 1, 5, 10 or 50 µM fucoidan 30 min prior to treatment with 25 µM erastin for 48 h Proteins were harvested for Western blotting. GPX4 levels were normalized to GAPDH. Concentration responses show biological replicates around the regression. Black lines indicate treatment of ARPE-19 or OMM-1 cells with increasing fucoidan concentrations without erastin treatment. Blue lines indicate treatment of ARPE-19 cells with increasing fucoidan concentrations and 20 µM erastin. GPX4 levels were normalized to GAPDH. Concentration responses show biological replicates around the regression. *n* = 4. Only descriptive data is presented due to the small sample size.

Next, we sought to determine whether the protective effect of FS, FE, and Fuc1 in ARPE-19 and OMM-1 cells was due to their ability to increase the protein expression of GPX4. GPX4 is an antioxidant defense enzyme that reduces lipid peroxides into lipid alcohols and is known to play a crucial role in ferroptosis [33]. As expected, erastin reduced GPX4 expression (vehicle normalized to 1.0) in both cell lines (Figure 9, median: 0.5 for ARPE-19 cells, 0.4 for OMM-1 cells). The different protective fucoidan extracts concentration-dependently attenuated the erastin-induced decrease. In ARPE-19 cells (Figure 9A), FS completely abrogated the GPX4 protein decrease starting at 10 µg/mL (median: 1.0), while Fuc1 treatment led to a partial recovery at 10–50 µg/mL (median: 0.7 for 10 µg/mL, 0.8 for 50 µg/mL). Concerning OMM-1 cells (Figure 9B), 50 µg/mL FE and Fuc1 increased GPX4 levels after erastin treatment (median: 0.7 for FE, 0.8 for Fuc1). However, the small sample size and large variation of the data were limitations of this study, and therefore, we did not perform statistical analyses. The effect of fucoidans on GPX4 should be confirmed in future studies.

## 3. Discussion

In this study, we characterized novel fucoidan extracts from different brown algae species and with different chemical properties. We demonstrated that they exert differential protective effects in ARPE-19 and OMM-1 cells exposed to iron-dependent oxidative stress induced by erastin (ferroptosis), while none of the assessed extracts was protective in neuronal ferroptosis. We further showed that this effect may be mediated by abrogating the decrease in GPX4 induced by erastin.

High-molecular weight fucoidans are described as antioxidative, antiangiogenic, and anti-inflammatory [34,35,36]. We have previously demonstrated that fucoidans from *Saccharina latissima*, *L. digitata*, *L. hyperborea*, *F. distichus subsp. evanescens*, *F. serratus*, and *F. vesiculosus* can exert antioxidative effects against H_2_O_2_ or tert-butylhydroperoxide (TBHP) depending on the cellular model system (ARPE-19, OMM-1), the used brown algae species, molecular weight, purity, and extraction procedure [4,5,37,38].

Here, we assessed iron-dependent oxidative stress (as induced by erastin leading to glutathione depletion, i.e., ferroptosis) because of its relevance in subretinal and brain hemorrhage as well as neurodegeneration. The overall protective effects of the tested fucoidans were limited. Only FS, FE, and Fuc1 showed partial protective effects against erastin-induced cell death in ARPE-19 (Figure 4) and OMM-1 cells (Figure 5). To note, we have previously used Fuc1, a high-molecular weight fucoidan that, besides a middle-sized and low-molecular weight *L. hyperborea* fucoidan, showed protective effects in OMM-1 cells against H_2_O_2_ after 24 h, but not in ARPE-19 cells against TBHP treatment for 24 h [4]. We also demonstrated that *F. vesiculosus* fucoidan from Sigma-Aldrich had protective properties in several uveal melanoma cell lines [39], which may depend on the fucoidan batch used. Another previous study from our group suggested that fucoidans from brown algae species *Saccharina latissima*, *L. digitate, F. distichus subsp. evanescens*, *F. serratus* and *F. vesiculosus* also seem promising for oxidative stress protection in OMM-1 (24 h) [38].

The overall biological activities are known to depend on the algae species and extraction procedure, but it is in fact their structural composition that determines their activities. Accordingly, the pronounced differences between the structural characteristics of the various *F. vesiculosus* fucoidans tested in this study could explain why all *F. vesiculosus* fucoidans in this study did not show any real antioxidative properties. We already showed before that the extraction procedure and purification steps, and thus the resulting structural characteristics, can influence the protective ability of the fucoidans [5]. Importantly, in the previous studies, higher H_2_O_2_ concentrations (1000 µM) were needed to exert 50% toxicity after 24 h in OMM-1 cells. We here decided to assess OMM-1 cells at 48 h using the half-maximal lethal dose of 250 µM H_2_O_2_, and hence the time of stimulation may have had an effect on the protective ability of the fucoidans as well. Future studies should also investigate whether repeated application of fucoidans may recover their protective effects.

What may be the reason for the difference in protective effects among the different extracts? Fucoidans are known for their heterogeneity, and many aspects could contribute to these differences: (1) All fucoidans contain mainly fucose, but the antioxidative properties seemed to be independent of the level of fucose [5]. Similarly, the levels of other monosaccharides were not indicative of the antioxidative potential of the fucoidan extracts. (2) The level of protein also did not seem to make an impact, as both Fuc1 and FVs lack protein, while Fuc1 was protective and FVs was not protective against erastin-induced cell death. (3) Except for the very high degree of sulfation of Fuc1 (1.7), this parameter was quite heterogenous for all other fucoidans, and thus we were not able to observe a direct correlation to oxidative defense capabilities. Sulfate groups contribute to scavenging free radicals, but a steric hindrance of the polymer chains results in long, coil-like molecule structures. In addition, low-molecular weight fucoidans are more capable of scavenging radicals because the sulfate and hydroxyl groups are more exposed due to the compact molecules [40]. (4) Regarding size, we have previously demonstrated that higher molecular weight fucoidans are more promising in terms of their ability to protect against H_2_O_2_-induced death [4]. In this study, the middle-sized FE (148 kDa) and FS (245 kDa) as well as the larger Fuc1 (1548 kDa) showed protective effects against erastin in ARPE-19 and OMM-1 cells, while other fucoidans of similar size did not. We also showed before that high-molecular weight fucoidans are more promising in terms of oxidative stress protection and may interact in cellular pathways and not reactive oxygen species scavenging [4,5,38]. In addition, the polydispersity of the extracts investigated in this study ranged from 1.5 (Fuc1) to 7.5 (FE). Because the extracts on both ends were slightly protective, polydispersity likely did not affect the protective potential in this model.

Overall, we were not able to find any plausible correlation to the structure data, extraction procedure, and chemical composition. As these are extracts and not pure compounds, the observed biological effect cannot be ascribed to a single structure. Each extract contains several fragments that may work synergistically or lead to compound–compound interactions. Future studies should assess different fractions of the extracts to give insight into the relationship between structure and biological activity.

Furthermore, it was remarkable that the tested fucoidan species were only partially protective in the ocular cell lines, while their protective effects were absent in neuronal cell lines and primary neurons. Ocular and neuronal cells show similar antioxidative defense mechanisms such as superoxide dismutase, glyoxalase glutathione reductase, glutathione peroxidase, catalase and nuclear factor E2-related factor 2 (Nrf2) [41,42]. However, RPE cells have developed strong antioxidant defense mechanisms to withstand the high amounts of reactive oxidative species due to light exposure and high metabolism rates [43]. Hence, higher concentrations of erastin and H_2_O_2_ may be needed to induce cell death in these cells. HT-22 cells are also known to be more sensitive to oxidative stress [41]. The main difference with uveal and RPE cells is the presence of melanin, which has antioxidative properties and can bind iron [44].

**Figure 10:**
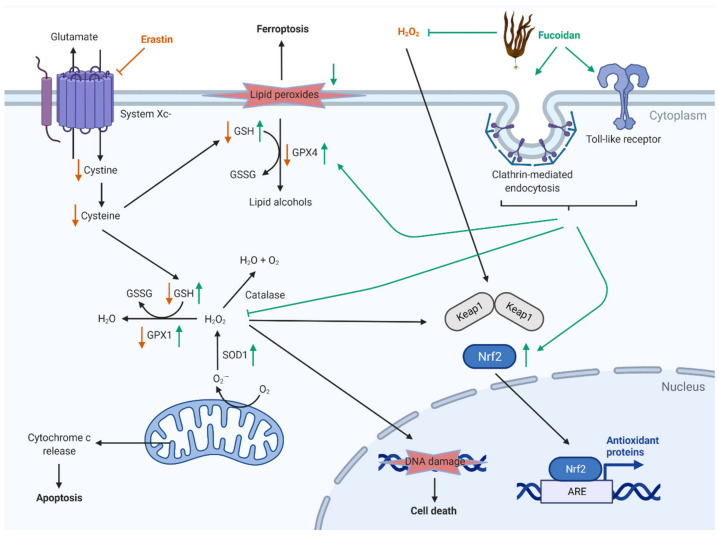
Potential mechanisms of action of fucoidans in oxidative stress. Figure based on references [45,46,47,48,49,50,51,52,53] and data of this study. Red arrows indicate the effect by erastin and H_2_O_2_. Green arrows indicate the effect by fucoidan. ARE—antioxidant responsive element, GPX—glutathione peroxidase, GSH—glutathione, GSSG—reduced glutathione, Nrf2—nuclear erythroid 2-related factor 2, SOD1—superoxide dismutase 1. Created with BioRender.com.

The possible interactions of fucoidans also depend on whether they are taken up by cells or only affect the cell from outside via scavenging oxidative reagents (such as H_2_O_2_) or interacting with membrane receptors (Figure 10). Fucoidans have been suggested to activate toll-like receptors [45]. Measurements of fucoidans intracellularly to demonstrate that they can enter cells remain technically challenging [46]. Of note, a fluorescently labeled fucoidan from *F. vesiculosus* was recently shown to be taken up into Caco-2 colorectal carcinoma cells via clathrin-mediated endocytosis [47]. Furthermore, FV fucoidan from Sigma-Aldrich has been demonstrated to increase the activity of the antioxidant enzyme superoxide dismutase 1 [48], and fucoidans (undefined source) augmented nuclear factor E2-related factor 2, a master regulator of gene expression induced by oxidative stress [49,50,51,52]. In a rat model of acetaminophen-induced liver injury, orally administered fucoidan from *F. vesiculosus* attenuated the decrease in glutathione, superoxide dismutase, and GPX, while blocking the increase in malondialdehyde [53], a marker of lipid peroxidation.

In the present study, we further add first evidence that fucoidans can abrogate the decrease in the protein levels of the antioxidant enzyme GPX4 that is crucial for ferroptosis. We plan to examine other ferroptosis markers such as reactive oxygen species production, lipid peroxidation, and glutathione levels in the future. Further studies are needed to determine the exact mechanisms by which fucoidans exert their protective effects against oxidative stress.

## 4. Material and Methods

### 4.1. Cell Lines

The human RPE cell line ARPE-19 [54] (ATCC, RRID: CVCL_0145) was cultured in 96-well plates (15,000 cells/well) in HyClone DMEM (GE Healthcare) containing 10% fetal calf serum (Linaris GmbH, Wertheim-Bettingen, Germany), 1% penicillin/streptomycin (Merck), 2.5% HEPES (Merck) and 1% non-essential amino acids (Merck). The uveal melanoma cell line OMM-1 (RRID: CVCL_6939) [55], provided by Dr. Sarah Coupland, was cultivated in 96-well plates (15,000 cells/well) in RPMI 1640 media (Merck), supplemented with 10% fetal calf serum (Linaris GmbH) and 1% penicillin/streptomycin (Merck). Immortalized hippocampal neuroblasts (HT-22 cells, RRID:CVCL_0321, Merck Millipore, Burlington, MA, USA) and SH-SY5Y cells (Depositor: JL Biedler, RRID:CVCL_0019, American Type Culture Collection (ATCC), Manassas, VA, USA) were cultured in 96-well plates in DMEM containing 10% fetal calf serum and 1% penicillin/streptomycin (4000 cells/well and 20,000 cells/well, respectively). Cell lines were treated 24 h after plating when the density reached at least 70% confluency. All cells were cultured at 37 °C in a humidified 5% CO_2_ atmosphere.

### 4.2. Primary Cortical Neurons

Primary cortical neurons were obtained from Crl:CD1 (ICR, Institute for Cancer Research, Philadelphia, PA, USA) Swiss outbred mice (Charles River Laboratories, Wilmington, MA, USA) of either sex at embryonic day 14.5. The animals were kept at 20–22 °C, 30–70% humidity in a 12-h/12-h light/dark cycle and were fed a standard chow diet (Altromin Spezialfutter GmbH & Co. KG, Lage, Germany) ad libitum. Animal experiments were performed in accordance with the German Animal Welfare Act and the corresponding regulations. Experimental procedures were approved by the local animal ethics committee (Ministerium für Landwirtschaft, Umwelt und ländliche Räume, Kiel, Germany, under the prospective contingent animal license number 2017-07-06 Zille).

We isolated primary cortical neurons from the embryos after decapitation as previously described [24]. We seeded the neurons (100,000 cells/well) in poly-d-lysine-coated (Sigma-Aldrich, St. Louis, MO, USA) 96-well plates in minimum essential medium (Thermo Fisher Scientific, Waltham, MA, USA) containing 10% fetal calf serum (Thermo Fisher Scientific), 5% horse serum (GE Healthcare, Chicago, IL, USA) and 1% penicillin/streptomycin (Merck KGaA, Darmstadt, Germany). Neurons were cultured at 37 °C in a humidified 5% CO_2_ atmosphere. The cells were treated 24 h after plating.

### 4.3. Fucoidan Extraction

Three commercially available fucoidans were used. FVs was purchased from Sigma-Aldrich, and FVm and UPm were provided by Marinova (Cambridge TAS, Australia).

All algae extracts obtained newly for this study were washed from saprophytes before drying. High-molecular weight *L. hyperborea* fucoidan Fuc1 (1548.6 kDa), identified and obtained from Alginor ASA and described in [4,56], was used. The extraction method and chemical data for molecular weight, sulfate and monosaccharide content as well as structure were described by Dörschmann et al. 2019 [4]. Three *Fucus* species, *F. vesiculosus* (FV), *F. serratus* (FS), and *F. evanescens* (FE), were identified and harvested by Coastal Research & Management in Kiel Fjord in Germany. The algae were soaked in an ethanol solution (85%) for 15 h. After soaking, the algae were carefully washed with acetone and left to dry for 4 h. Once dried, the algae were ground into 1 mm particles and the fucoidans were prepared as follows:

For FV1, *F. vesiculosus* (harvested in July 2017) was submerged in 100 mM hydrochloric acid (ambient temperature) for 24 h, decanting the acid and subsequently neutralizing it with 1 M sodium hydroxide. The neutralized extraction solvent was transferred to a tube and aqueous calcium chloride (35%) was added in amounts corresponding to 1% calcium chloride in the extraction solvent. The solution was centrifuged for 30 min and the resulting supernatant was recovered. Ethanol was added to the supernatant in amounts corresponding to a 40% ethanol concentration. The solution was centrifuged for another 30 min, and the supernatant was recovered again. Ethanol was added to a final concentration of 70% ethanol, and the solution was centrifuged again. The pellet was collected and washed with ethanol and acetone to dry. Next, the fucoidan pellet was solubilized in deionized water and dialyzed (MWCO = 12–14 kDa), until the conductivity of the deionized water remained constant. The fucoidan was subsequently freeze-dried prior to chemical and biological analysis.

FV2 fucoidan was prepared from the same algae used for FV1 fucoidan, using similar extraction conditions; however, the algae were submerged in hydrochloric acid four times for 24 h, with a decanting and fresh addition of hydrochloric acid after every 24 h. After 96 h, the acid was decanted and neutralized with 1 M sodium hydroxide and subjected to the same cleanup procedure as FV1.

The fucoidans FV3, FS and FE (harvested in October 2017) were prepared by adding 1.5 g of algae material and 100 mM hydrochloric acid to a microwave extraction vessel, which was heated at 80 °C for 30 min in a microwave digestion system (Multiwave GO, Anton Paar). After cooling, the extraction solvent was neutralized with 1 M sodium hydroxide. All fucoidans were recovered and purified by calcium chloride and ethanol, as described for FV1.

Endotoxin levels were not measured in the extracts used in this study. However, microwave irradiation and exposure to acids degrade endotoxins [57,58]. In addition, FVs had been previously shown to be ultra-pure with respect to endotoxins (below 100 EU/mL) [59]. In general, we considered all extracts to be endotoxin-free.

All fucoidans were dissolved in aqua bidest, stored at −20 °C and sterile filtrated before use.

### 4.4. Size Exclusion Chromatography with Multiple Detection

We determined the molecular weight, size, and chain conformation of the fucoidan samples using size-exclusion chromatography coupled with multiple detection. The instrument setup consisted of an Agilent 1200 series HPLC (Agilent Technologies, Waldbronn, Germany) connected to two OHPak LB-806M size-exclusion chromatography columns (8.0 mmID × 300 mmL) and a guard column (Shodex, Munich, Germany). The chromatographically separated samples were detected using an Agilent 1200 series UV detector, a DAWN™ MALS photometer, a ViscoStar^®^ on-line differential viscometer and an Optilab^®^ differential refractometer (Wyatt Technology, Dernbach, Germany) connected in series. The samples were eluted using a Na_2_HPO_4_–NaH_2_PO_4_ (50 mM) buffer solution (Ph 7.0) containing 150 mM NaCl. The samples (2 mg/mL) were filtered using a 0.45 µm filter before injection to remove any large particles. The used dn/dc was 0.150 mL/g for each fucoidan. Data acquisition and analysis were performed using ASTRA^®^ 8.0 software (Wyatt Technology, Dernbach, Germany).

### 4.5. Elemental Analysis

The sulfur and nitrogen contents of the fucoidan samples were determined by elemental analysis as previously described [60]. The percentage of sulfate groups (calculated as –SO_3_Na) was used to calculate the degree of sulfation. The total protein content was estimated by multiplying the nitrogen content (%) by 6.25.

### 4.6. Monosaccharide Composition by Gas-Liquid Chromatography of Alditol Acetates

We determined the neutral monosaccharide composition of the fucoidans by acetylation analysis as previously described [60]. Briefly, the fucoidans were hydrolyzed using trifluoroacetic acid [61], reduced and acetylated to obtain alditol acetate derivatives (AA) [62]. The AA were separated using gas-liquid chromatography, and the percentage of the respective AA were calculated as previously reported [37].

### 4.7. Treatments

First, different concentrations of erastin (0.1–30 µM, Cayman Chemical, Ann Arbor, MI, USA) and H_2_O_2_ (100–1000 µM, Sigma-Aldrich) diluted in cell culture medium were tested to determine the half-maximal lethal dose at 24 or 48 h, depending on the cell type. Next, the protective effect of fucoidan on erastin- or H_2_O_2_-induced oxidative stress at half-maximal lethal dose was assessed. Therefore, we added erastin or H_2_O_2_ 30 min after fucoidan treatment and incubated the cells for 24 or 48 h. For primary cortical neurons, fucoidan concentrations of 0–5 μg/mL were used because higher concentrations led to cell detachment. For all cell lines, fucoidan concentrations between 0 and 50 μg/mL were applied. Ferrostatin-1 (Sigma-Aldrich) was used as a positive control for erastin-induced oxidative stress in neurons and was incubated as described for fucoidan.

### 4.8. Cell Viability

We determined the cell viability 24 h after erastin or H_2_O_2_ exposure, except for OMM-1 cells that were assessed at 48 h because 24 h were not sufficient to kill the cells with erastin. We used the 1-(4,5-dimethylthiazol-2-yl)-3,5-diphenylformazan (MTT, Carl Roth GmbH + Co. KG, Karlsruhe, Germany) assay, a colorimetric assay of cell metabolic activity [63]. The water-soluble tetrazolium salt is reduced by metabolically active cells to water-insoluble formazan. After 2–4 h of incubation depending on the cell type, MTT is removed and the cells are permeabilized with dimethylsulfoxide to visualize the formazan crystals. We measured the plates with a CLARIOstar Microplate Reader using CLARIOstar v.5.20 R5/MARS v.3.10 R5 (all BMG LABTECH, Ortenberg, Germany) or Elx800 microplate reader from BioTek (Bad Friedrichshall, Germany). The maximum extinction of formazan is at 550 nm, but at the same wavelength, the dissolvent dimethyl sulfoxide has an extinction of 0.03 OD. Therefore, the wavelength at 655 nm was also measured and subsequently subtracted from the OD value at 550 nm. The mean of four technical replicates was calculated for all conditions and subsequently normalized to the mean of the vehicle treatment without addition of erastin (representing one biological replicate).

The results of the population, quantitative assays of cell viability (MTT) were verified qualitatively using calcein acetoxymethyl (calcein AM, 2 µM, Santa Cruz Biotechnology, Dallas, TX, USA) and propidium iodide (3 µM, Carl Roth) incubated for 15–30 min directly in the cell culture medium without medium change. Calcein AM is cell-permeable and is cleaved by esterases in intact cells. The calcein can then bind to calcium ions, and this binding results in green fluorescence staining [64]. Propidium iodide is a nucleic staining dye that is not able to pass through a viable cell membrane, but can only pass disturbed cell membranes of damaged or dead cells. It interacts with the DNA double helix and emits red fluorescence [65]. Pictures were taken with an Axiovert 200 M or Axiovert 100 microscope (Carl Zeiss AG, Oberkochen, Germany).

### 4.9. Western Blot

GPX4 expression was assessed with Western blot. After stimulating ARPE-19 and OMM-1 cells with erastin for 24 h or 48 h, respectively, cell lysates were prepared. Cells were washed with ice-cold PBS (Merck), scratched off in and lysed with radioimmunoprecipitation assay buffer (RIPA, Sigma Aldrich) containing 150 mM NaCl, 1% NP-40, 0.5% sodium deoxycholate, 0.1% SDS (sodium dodecyl sulfate), 50 mM Tris-HCl (pH 8.0), 1% protease inhibitor cocktail (Sigma-Aldrich) and 1% of each phosphatase inhibitor cocktail 1 & 2 (Sigma-Aldrich) for 25 min at 4 °C. The lysates were centrifuged at 12,000 rpm for 20 min and the supernatant with proteins was photometrically analyzed with Bio-Rad protein assay (Bio-Rad) to measure protein concentrations. Both SDS-PAGE and Western blot were conducted as described before [12,66]. Thirty micrograms of proteins were separated with SDS-PAGE (SDS polyacrylamide gel electrophoresis) (12% gel) and gels were used in wet tank blot. Blocking for 1 h was conducted with 2% dry milk (Carl Roth GmbH + Co. KG) in Tris-buffered saline plus Tween (TBST) (Merck). Primary antibodies rabbit anti-GPX4 (1:1000, abcam, cat. no. ab125066, Cambridge, United Kingdom) and rabbit anti-GAPDH (1:1000, cat. no. 2118, Cell Signaling Technologies, Danvers, MA, USA) were incubated overnight at 4 °C. Anti-rabbit horseradish peroxidase conjugates were used for detection (1:1000, Cell Signaling Technologies), after adding ECL (Enhanced Chemiluminescence) Western Blotting Detection Reagent (GE Healthcare). The signal was measured with MF-ChemiBis 1.6 (Biostep, Jahnsdorf, Germany) and photos of the bands were taken for qualitative description.

### 4.10. Statistical Analysis

Normality was evaluated by the Kolmogorov–Smirnov test and variance homogeneity using Levené test. Because data were not normally distributed or variance homogeneity was not met, Kruskal–Wallis test was performed followed by the post hoc Mann–Whitney U test with α-correction according to Bonferroni–Holm to adjust for the inflation of type I error due to multiple testing. *p* < 0.05 was considered statistically significant. Vehicle was compared to erastin or H_2_O_2_ and the individual fucoidan concentrations with erastin or H_2_O_2_ were compared to erastin or H_2_O_2_ alone. Each biological replicate was displayed around the regression (concentration-response curves) or median (ferrostatin-1). All statistical analyses were performed with IBM SPSS version 23.

## 5. Conclusions

The aim of this study was to investigate different high-molecular weight fucoidan extracts from brown seaweed of different origins for application against iron-dependent oxidative stress, a main hallmark of retinal and brain diseases, including retinal and brain hemorrhage. Only extracts from *F. serratus*, *F. distichus* subsp. *evanescens* and *Laminaria hyperborea* were partially protective against erastin-induced cell death in ARPE-19 and OMM-1 cells, while none of the extracts showed beneficial effects in neuronal cells. Preliminary data suggest that the protective effects may be linked to increasing GPX4 protein levels, a key antioxidant enzyme involved in iron-dependent oxidative stress, i.e., ferroptosis. Our comprehensive analysis suggests that the antioxidant abilities of fucoidans may be cell type-specific in addition to depending on the algal species and extraction method. Future studies are needed to further characterize the health-benefiting effects of fucoidans and to determine the exact mechanism underlying their antioxidative abilities.

## Figures and Tables

**Table 1:** Composition of neutral monosaccharides, degree of sulfation, and protein content in the extracted fucoidans.

	Monosaccharide Composition (mol %) ^a^					Degree of Sulfation ^b^	Protein Content (%) ^c^
	Fuc	Xyl	Man	Gal	Glc		
FV1	50.5	7.3	2.7	5.3	34.2	0.32	0.42
FV2	67.2	19.8	2.3	6.3	4.3	0.42	6.20
FV3	80.7	4.3	2.6	7.5	4.9	0.26	2.16
FS	77.2	5.8	1.8	4.2	11.0	0.35	2.04
FE	87.1	3.6	2.1	6.7	0.5	0.45	4.88
FVs	86.2	4.1	1.6	4.4	0.6	0.61	0.00
Fuc1 ^d^	97.0	0.0	0.0	3.0	0.0	1.70	0.00

^a^ Determined according to the method of Blakeney et al. (1983) [27]. ^b^ Averaged number of sulfate groups per monosaccharide, calculated as -SO_3_Na residues (FV1: 18.3%, FV2: 22.9%, FV3: 15.6%, FS: 19.8%, FE: 24.1%) from sulfur content determined by elemental analysis. ^c^ The protein content was calculated by elemental analysis (nitrogen (%)). ^d^ From Dörschmann et al. (2019) [4].

**Table 2:** Molecular mass and size characteristics of the fucoidans with number-average molar mass (Mn), molecular weight (M_W_), polydispersity (PD; M_W_/Mn), rms radius (rg), hydrodynamic radius (rh), and intrinsic viscosity ([ŋ]).

	MW (kDa)	Mn (kDa)	PD (MW/Mn)	rg (nm)	rh (nm)	[η] (mL/g)
**FV1**	261	33	8.0	27.9	28.8	61.2
**FV2**	1438	337	4.3	31.3	38.7	78.8
**FV3**	339	103	3.3	36.8	34.9	94.0
**FS**	245	64	3.8	32.0	26.5	73.5
**FE**	148	20	7.5	26.5	21.7	73.7
**FVs** **Fuc1 ^a^**	52 1548	34 1021	1.5 1.5	n.a. n.a.	6.9 n.a.	19.8 n.a.

^a^ From Dörschmann et al. (2019) [4].

**Table 3:** Summary of the effects of fucoidan extracts. Cells were exposed to fucoidan 30 min prior to treatment with erastin or H_2_O_2_ for 24 or 48 h. Values represent medians of 50 µg/mL fucoidan treatment with erastin, except for primary cortical neurons (PCN), where 5 µg/mL fucoidan was used. * *p* < 0.05 compared to vehicle treatment without erastin or H_2_O_2_. # *p* < 0.05 compared to erastin plus vehicle. *n* = 6–9. For concentration responses, refer to Figure 4, Figure 5, Figure 6, Figure 7 and Figure 8.

Treatment	ARPE-19, Erastin 24 h	ARPE-19, H_2_O_2_ 24 h	OMM-1, Erastin 48 h	OMM-1, H_2_O_2_ 48 h	HT-22, Erastin 24 h	SH-SY5Y, Erastin 24 h	PCN, Erastin 24 h
Vehicle	63.59 *	61.53 *	34.73 *	38.33 *	33.96 *	43.24 *	41.77 *
FVs	56.88	41.70	60.71	36.62	30.98	44.97	35.61
FVm	60.32	42.41	37.37	44.50	29.58	54.47	43.10
UPm	63.49	41.17	70.22	29.41	29.68	57.65	34.29
FV1	44.30	55.83	50.44	29.54	33.79	50.17	39.43
FV2	33.61	46.38	33.18	46.17	35.33	27.09	44.88
FV3	47.99	58.85	58.06	33.93	20.92	31.33	33.13
FS	85.71 #	74.65	55.31	33.17	18.32	32.10	38.53
FE	80.47 #	56.00	56.17 #	26.50	28.11	54.69	46.77
Fuc1	81.15	57.35	51.60	48.64	29.83	50.98	68.35

## Data Availability

Data on fucoidans is available at Zenodo data base (doi.org/10.5281/zenodo.3876379, accessed on 17 September 2021) and further data is available upon request.

## Supplementary Materials

The following are available online at: www.mdpi.com/1405679/s1, Table S1: Detailed statistical analyses of erastin and H_2_O_2_ concentration-responses, Table S2: Detailed statistical analyses of ARPE-19 cells exposed to 20 µM erastin and fucoidan for 24 h, Table S3: Detailed statistical analyses of ARPE-19 cells exposed to 500 µM H_2_O_2_ and fucoidan for 24 h, Table S4: Detailed statistical analyses of OMM-1 cells exposed to 25 µM erastin and fucoidan for 48 h, Table S5: Detailed statistical analyses of OMM-1 cells exposed to 250 µM H_2_O_2_ and fucoidan for 48 h, Table S6: Detailed statistical analyses of HT-22 cells exposed to 0.35 µM erastin and fucoidan for 24 h, Table S7: Detailed statistical analyses of SH-SY5Y cells exposed to 30 µM erastin and fucoidan for 24 h, Table S8: Detailed statistical analyses of primary cortical neurons exposed to 0.5 µM erastin and fucoidan for 24 h.
