# Supplementary material for: Evaluation of the Effects of Fucoidans from Fucus Species and Laminaria hyperborea against Oxidative Stress and Iron-Dependent Cell Death
Source: Mar Drugs. 2021 Sep 29;19(10):557. doi: 10.3390/md19100557 (PMC8538076; doi:10.3390/md19100557)
Supplement: Supplementary file 1 [file marinedrugs-19-00557-s001.zip › marinedrugs-1405679-supplementary.pdf]

marine drugs

Supplemental data

Evaluation of the Effects of Fucoidans from *Fucus* Species and *Laminaria hyperborea* Against Oxidative Stress and Iron-dependent Cell Death

Philipp Dörschmann [1,], Sarah Apitz [1], Inga Hellige [2,3], Sandesh Neupane [4], Susanne Alban [4], Georg Kopplin [5], Signe Ptak [6], Xavier Fretté [6], Johann Roider [1], Marietta Zille [2,3,7,†] and Alexa Klettner [1,*,†]

1 Department of Ophthalmology, University of Kiel, University Medical Center, Arnold-Heller-Str. 3, Haus 25, 24105 Kiel, Germany; Philipp.Doerschmann@uksh.de (P.D.); s-apitz@web.de (S.Ap.); Johann.Roider@uksh.de (J.R.); Alexa.Klettner@uksh.de (A.K.)
2 Fraunhofer Research and Development Center for Marine and Cellular Biotechnology EMB, Mönkhofer Weg 239a, 23562 Lübeck, Germany; inga.hellige@t-online.de (I.H.); m.zille@uni-luebeck.de (M.Z.)
3 Institute for Medical and Marine Biotechnology, University of Lübeck, Mönkhofer Weg 239a, 23562 Lübeck, Germany;
4 Pharmaceutical Institute, Kiel University, Gutenbergstraße 76, 24118 Kiel, Germany; sneupane@pharmazie.uni-kiel.de (S.N.); salban@pharmazie.uni-kiel.de (S.Al.)
5 Alginor ASA, Haraldsgata 162, 5525 Haugesund, Norway, E-mail: Georg@alginor.no
6 University of Southern Denmark, Campusvej 55, 5230 Odense, Denmark; sihp@igt.sdu.dk (S.P.); xafr@kbm.sdu.dk (X.F.)
7 Department of Pharmaceutical Sciences, Division of Pharmacology and Toxicology, University of Vienna, UZA II, Althanstraße 14, 1090 Vienna, Austria; marietta.zille@univie.ac.at
* Correspondence: Alexa.Klettner@uksh.de, Tel: 0049-431-500-24283
† These authors contributed equally to the work.

marine drugs

MDPI

Table S1. Detailed statistical analyses of erastin and H_2O_2 concentration-responses.

	Kolmogorov-Smirnov test	Levené test	Kruskal-Wallis test	Post hoc Mann-Whitney U with Bonferroni-Holm correction
ARPE-19 cells, erastin 24 hours	Z = 0.100, *P* = 0.200	F(5,36) = 6.158, *P* < 0.001	[chi]2(5,N = 42) = 15.846, *P* = 0.007, η^2 = 0.386	*P* = 0.633 for 5 µM, *P* = 0.004 for 10 µM, *P* = 0.009 for 15 µM, *P* = 0.004 for 20 µM, *P* = 0.034 for 25 µM erastin vs. vehicle
ARPE-19 cells, H_2O_2 24 hours	Z = 0.148, *P* = 0.028	F(4,35) = 11.384, *P* < 0.001	[chi]2(4,N = 40) = 20.823, *P* < 0.001, η^2 = 0.534	*P* = 0.001 for 100 µM, *P* = 0.007 for 250 µM, *P* = 0.001 for 500-1000 µM H_2O_2 vs. vehicle
OMM-1, erastin 48 hours	Z = 0.101, *P* = 0.200	F(6,35) = 4.067, *P* = 0.003	[chi]2(6,N = 42) = 25.305, *P* < 0.001, η^2 = 0.690	*P* = 1.000 for 5-20 µM, *P* = 0.012 for 25-30 µM erastin vs. vehicle
OMM-1, H_2O_2 48 hours	Z = 0.128, *P* = 0.200	F(4,25) = 10.237, *P* < 0.001	[chi]2(4,N = 30) = 26.963, *P* < 0.001, η^2 = 0.930	*P* = 0.008 for 100-1000 µM H_2O_2 vs. vehicle
HT-22 cells, erastin 24 hours	Z = 0.169, *P* = 0.001	F(7,42) = 14.274, *P* < 0.001	[chi]2(7,N = 49) = 40.251, *P* < 0.001, η^2 = 0.839	*P* = 1.000 for 0.1-0.2 µM, *P* = 0.005 for 0.3-1 µM erastin vs. vehicle
SH-SY5Y cells, erastin 24 hours	Z = 0.106, *P* = 0.174	F(6,49) = 5.270, *P* < 0.001	[chi]2(6,N = 56) = 42.423, *P* < 0.001, η^2 = 0.771	*P* = 0.002 for 5-10 µM, *P* = 0.007 for 15 µM, *P* = 0.002 for 20-30 µM erastin vs. vehicle
Primary neurons, erastin 24 hours	Z = 0.111, *P* = 0.200	F(4,20) = 1.953, *P* = 0.141	[chi]2(4,N = 25) = 21.522, *P* < 0.001, η^2 = 0.897	*P* = 0.021 for 0.2-1 µM erastin vs. vehicle

Table S2. Detailed statistical analyses of ARPE-19 cells exposed to 20 µM erastin and fucoidan for 24 hours.

	Kolmogorov-Smirnov test	Levené test	Kruskal-Wallis test	Post hoc Mann-Whitney U with Bonferroni-Holm correction
FVs	Z = 0.132, *P* = 0.007	F(9,55) = 3.177, *P* = 0.004	[chi]2(9,*N* = 65) = 46.782, *P* < 0.001, η^2 = 0.731	20 µM erastin vs. control: *P* = 0.008<br>20 µM erastin + fucoidan vs. 20 µM erastin:<br>1-50 µg/ml, *P* = 1.000
FVm	Z = 0.111, *P* = 0.045	F(9,55) = 1.705, *P* = 0.110	[chi]2(9,*N* = 65) = 45.719, *P* < 0.001, η^2 = 0.714	20 µM erastin vs. control: *P* = 0.008<br>20 µM erastin + fucoidan vs. 20 µM erastin:<br>1-50 µg/ml, *P* = 1.000
UPm	Z = 0.112, *P* = 0.041	F(9,55) = 3.661, *P* = 0.001	[chi]2(9,*N* = 65) = 40.794, *P* < 0.001, η^2 = 0.637	20 µM erastin vs. control: *P* = 0.008<br>20 µM erastin + fucoidan vs. 20 µM erastin:<br>1 µg/ml: *P* = 0.567, 5-50 µg/ml: *P* = 1.000
FV1	Z = 0.181, *P* < 0.001	F(9,55) = 3.322, *P* = 0.003	[chi]2(9,*N* = 65) = 35.555, *P* < 0.001, η^2 = 0.556	20 µM erastin vs. control: *P* = 0.008<br>20 µM erastin + fucoidan vs. 20 µM erastin:<br>1-50 µg/ml, *P* = 1.000
FV2	Z = 0.122, *P* = 0.017	F(9,55) = 3.492, *P* = 0.002	[chi]2(9,*N* = 65) = 37.982, *P* < 0.001, η^2 = 0.593	20 µM erastin vs. control: *P* = 0.008<br>20 µM erastin + fucoidan vs. 20 µM erastin:<br>1-50 µg/ml, *P* = 0.899
FV3	Z = 0.213, *P* < 0.001	F(9,55) = 3.374, *P* = 0.002	[chi]2(9,*N* = 65) = 38.937, *P* < 0.001, η^2 = 0.608	20 µM erastin vs. control: *P* = 0.008<br>20 µM erastin + fucoidan vs. 20 µM erastin:<br>1 µg/ml: *P* = 0.567, 5-50 µg/ml: *P* = 1.000
FS	Z = 0.105, *P* = 0.099	F(9,50) = 2.981,	[chi]2(9,*N* = 60) = 36.363, *P* < 0.001,	20 µM erastin vs. control: *P* = 0.008

		P = 0.006	$\eta^2 = 0.616$	20 μM erastin + fucoidan vs. 20 μM erastin:<br>1 μg/ml: *P* = 0.109, 5 μg/ml: *P* = 0.049, 10 μg/ml: *P* = 0.075,<br>50 μg/ml: *P* = 0.016
FE	Z = 0.101, *P* = 0.200	F(9,50) = 6.172,<br>*P* < 0.001	[chi]2(9,*N* = 60) = 47.236, *P* < 0.001,<br>$\eta^2 = 0.801$	20 μM erastin vs. control: *P* = 0.008<br>20 μM erastin + fucoidan vs. 20 μM erastin:<br>1 μg/ml: *P* = 0.423, 5 μg/ml: *P* = 0.109, 10 μg/ml: *P* = 0.075,<br>50 μg/ml: *P* = 0.026
Fuc1	Z = 0.114, *P* = 0.050	F(9,50) = 2.461,<br>*P* = 0.021	[chi]2(9,*N* = 60) = 47.516, *P* < 0.001,<br>$\eta^2 = 0.805$	20 μM erastin vs. control: *P* = 0.008<br>20 μM erastin + fucoidan vs. 20 μM erastin:<br>1-5 μg/ml: *P* = 0.400, 10 μg/ml: *P* = 0.100, 50 μg/ml, *P* = 0.164

Table S3. Detailed statistical analyses of ARPE-19 cells exposed to 500 μM H_2O_2 and fucoidan for 24 hours.

	Kolmogorov-Smirnov test	Levené test	Kruskal-Wallis test	Post hoc Mann-Whitney U with Bonferroni-Holm correction
FVs	Z = 0.199, *P* < 0.001	F(9,50) = 3.572, *P* = 0.002	[chi]2(9,*N* = 60) = 45.165, *P* < 0.001, η^2 = 0.766	500 μM H_2O_2 vs. control: *P* = 0.010<br>500 μM H_2O_2 + fucoidan vs. 500 μM H_2O_2:<br>1-50 μg/ml: *P* = 1.000
FVm	Z = 0.216, *P* < 0.001	F(9,50) = 2.561, *P* = 0.017	[chi]2(9,*N* = 60) = 47.020, *P* < 0.001, η^2 = 0.797	500 μM H_2O_2 vs. control: *P* = 0.010<br>500 μM H_2O_2 + fucoidan vs. 500 μM H_2O_2:<br>1 μg/ml: *P* = 1.000, 5 μg/ml: *P* = 0.313, 10 μg/ml: *P* = 0.449, 50 μg/ml: *P* = 1.000
UPm	Z = 0.154, *P* < 0.001	F(9,50) = 4.056, *P* = 0.001	[chi]2(9,*N* = 60) = 46.497, *P* < 0.001, η^2 = 0.788	500 μM H_2O_2 vs. control: *P* = 0.010<br>500 μM H_2O_2 + fucoidan vs. 500 μM H_2O_2:<br>1 μg/ml: *P* = 1.000, 5 μg/ml: *P* = 0.313, 10-50 μg/ml: *P* = 1.000
FV1	Z = 0.201, *P* < 0.001	F(9,50) = 2.908, *P* = 0.008	[chi]2(9,*N* = 60) = 43.350, *P* < 0.001, η^2 = 0.735	500 μM H_2O_2 vs. control: *P* = 0.010<br>500 μM H_2O_2 + fucoidan vs. 500 μM H_2O_2:<br>1 μg/ml: *P* = 0.749, 5 μg/ml: *P* = 0.345, 10 μg/ml: *P* = 0.164, 50 μg/ml: *P* = 0.100
FV2	Z = 0.145, *P* = 0.003	F(9,50) = 3.134, *P* = 0.005	[chi]2(9,*N* = 60) = 45.944, *P* < 0.001, η^2 = 0.779	500 μM H_2O_2 vs. control: *P* = 0.010<br>500 μM H_2O_2 + fucoidan vs. 500 μM H_2O_2:<br>1-50 μg/ml: *P* = 1.000
FV3	Z = 0.198, *P* < 0.001	F(9,50) = 2.425, *P* = 0.023	[chi]2(9,*N* = 60) = 44.866, *P* < 0.001, η^2 = 0.760	500 μM H_2O_2 vs. control: *P* = 0.010<br>500 μM H_2O_2 + fucoidan vs. 500 μM H_2O_2:

				1-50 μg/ml: $P = 1.000$
FS	Z = 0.163, $P < 0.001$	F(9,50) = 3.219, $P = 0.004$	[chi]2(9,N = 60) = 45.096, $P < 0.001$, $\eta^2 = 0.764$	500 μM H_2O_2 vs. control: $P = 0.010$<br>500 μM H_2O_2 + fucoidan vs. 500 μM H_2O_2:<br>1 μg/ml: $P = 0.601$, 5 μg/ml: $P = 0.598$, 10-50 μg/ml: $P = 1.000$
FE	Z = 0.085, $P = 0.200$	F(9,50) = 4.539, $P < 0.001$	[chi]2(9,N = 60) = 47.391, $P < 0.001$, $\eta^2 = 0.803$	500 μM H_2O_2 vs. control: $P = 0.010$<br>500 μM H_2O_2 + fucoidan vs. 500 μM H_2O_2:<br>1-10 μg/ml: $P = 1.000$, 50 μg/ml: $P = 0.065$
Fuc1	Z = 0.139, $P = 0.005$	F(9,50) = 2.693, $P = 0.012$	[chi]2(9,N = 60) = 47.522, $P < 0.001$, $\eta^2 = 0.805$	500 μM H_2O_2 vs. control: $P = 0.010$<br>500 μM H_2O_2 + fucoidan vs. 500 μM H_2O_2:<br>1-5 μg/ml: $P = 0.328$, 10 μg/ml: $P = 0.337$, 50 μg/ml: $P = 0.219$

Table S4. Detailed statistical analyses of OMM-1 cells exposed to 25 μM erastin and fucoidan for 48 hours.

	Kolmogorov-Smirnov test	Levené test	Kruskal-Wallis test	Post hoc Mann-Whitney U with Bonferroni-Holm correction
FVs	Z = 0.131, *P* = 0.005	F(9,60) = 4.144, *P* < 0.001	[chi]2(9,*N* = 70) = 54.176, *P* < 0.001, η^2 = 0.785	25 μM erastin vs. control: *P* = 0.004<br>25 μM erastin + fucoidan vs. 25 μM erastin:<br>1 μg/ml: *P* = 0.072, 5 μg/ml: *P* = 0.105, 10 μg/ml: *P* = 0.225, 50 μg/ml: *P* = 0.128
FVm	Z = 0.183, *P* < 0.001	F(9,50) = 5.551, *P* < 0.001	[chi]2(9,*N* = 60) = 49.615, *P* < 0.001, η^2 = 0.841	25 μM erastin vs. control: *P* = 0.010<br>25 μM erastin + fucoidan vs. 25 μM erastin:<br>1-5 μg/ml: *P* = 0.065, 10 μg/ml: *P* = 0.525, 50 μg/ml: *P* = 0.749
UPm	Z = 0.190, *P* < 0.001	F(9,60) = 3.473, *P* = 0.002	[chi]2(9,*N* = 70) = 53.145, *P* < 0.001, η^2 = 0.770	25 μM erastin vs. control: *P* = 0.004<br>25 μM erastin + fucoidan vs. 25 μM erastin:<br>1-10 μg/ml: *P* = 1.000, 50 μg/ml, *P* = 0.072
FV1	Z = 0.197, *P* < 0.001	F(9,80) = 3.723, *P* < 0.001	[chi]2(9,*N* = 90) = 69.130, *P* < 0.001, η^2 = 0.777	25 μM erastin vs. control: *P* < 0.001<br>25 μM erastin + fucoidan vs. 25 μM erastin:<br>1 μg/ml: *P* = 0.825, 5 μg/ml: *P* = 0.648, 10 μg/ml: *P* = 0.340, 50 μg/ml, *P* = 0.648
FV2	Z = 0.229, *P* < 0.001	F(9,70) = 3.881, *P* < 0.001	[chi]2(9,*N* = 80) = 63.329, *P* < 0.001, η^2 = 0.802	25 μM erastin vs. control: *P* = 0.002<br>25 μM erastin + fucoidan vs. 25 μM erastin:<br>1-10 μg/ml: *P* = 0.345, 50 μg/ml: *P* = 0.063
FV3	Z = 0.197, *P* < 0.001	F(9,70) = 7.136, *P* < 0.001	[chi]2(9,*N* = 80) = 62.196, *P* < 0.001, η^2 = 0.787	25 μM erastin vs. control: *P* = 0.002<br>25 μM erastin + fucoidan vs. 25 μM erastin:

				1-50 μg/ml: *P* = 0.992
FS	Z = 0.173, *P* < 0.001	F(9,60) = 2.994,<br>*P* = 0.005	[chi]2(9,*N* = 70) = 58.165, *P* < 0.001,<br>η^2 = 0.868	25 μM erastin vs. control: *P* = 0.004<br>25 μM erastin + fucoidan vs. 25 μM erastin:<br>1 μg/ml: *P* = 0.812, 5 μg/ml: *P* = 0.478, 10 μg/ml: *P* = 0.812, 50 μg/ml,<br>*P* = 0.101
FE	Z = 0.194, *P* < 0.001	F(9,50) = 3.602,<br>*P* = 0.002	[chi]2(9,*N* = 60) = 52.338, *P* < 0.001,<br>η^2 = 0.887	25 μM erastin vs. control: *P* = 0.010<br>25 μM erastin + fucoidan vs. 25 μM erastin:<br>1 μg/ml: *P* = 0.109, 5 μg/ml: *P* = 0.019, 10 μg/ml: *P* = 0.021, 50 μg/ml,<br>*P* = 0.016
Fuc1	Z = 0.149, *P* = 0.002	F(9,50) = 4.641,<br>*P* < 0.001	[chi]2(9,*N* = 60) = 47.508, *P* < 0.001,<br>η^2 = 0.805	25 μM erastin vs. control: *P* = 0.010<br>25 μM erastin + fucoidan vs. 25 μM erastin:<br>1 μg/ml: *P* = 0.337, 5 μg/ml: *P* = 0.112, 10 μg/ml: *P* = 0.026, 50 μg/ml,<br>*P* = 0.112

Table S5. Detailed statistical analyses of OMM-1 cells exposed to 250 µM H_2O_2 and fucoidan for 48 hours.

	Kolmogorov-Smirnov test	Levené test	Kruskal-Wallis test	Post hoc Mann-Whitney U with Bonferroni correction at α = 0.01
FVs	Z = 0.172, *P* < 0.001	F(9,60) = 2.931, *P* = 0.006	[chi]2(9,*N* = 70) = 53.978, *P* < 0.001, η^2 = 0.782	250 µM H_2O_2 vs. control: *P* = 0.004<br>250 µM H_2O_2 + fucoidan vs. 250 µM H_2O_2:<br>1 µg/ml: *P* = 0.035, 5-50 µg/ml: *P* = 0.539
FVm	Z = 0.172, *P* < 0.001	F(9,50) = 4.671, *P* < 0.001	[chi]2(9,*N* = 60) = 43.561, *P* < 0.001, η^2 = 0.738	250 µM H_2O_2 vs. control: *P* = 0.010<br>250 µM H_2O_2 + fucoidan vs. 250 µM H_2O_2:<br>1-50 µg/ml: *P* = 1.000
UPm	Z = 0.231, *P* < 0.001	F(9,60) = 2.652, *P* = 0.012	[chi]2(9,*N* = 70) = 54.380, *P* < 0.001, η^2 = 0.788	250 µM H_2O_2 vs. control: *P* = 0.004<br>250 µM H_2O_2 + fucoidan vs. 250 µM H_2O_2:<br>1 µg/ml: *P* = 0.283, 5 µg/ml: *P* = 0.051, 10 µg/ml: *P* = 0.749, 50 µg/ml, *P* = 0.191
FV1	Z = 0.218, *P* < 0.001	F(9,80) = 8.801, *P* < 0.001	[chi]2(9,*N* = 90) = 67.971, *P* < 0.001, η^2 = 0.764	250 µM H_2O_2 vs. control: *P* < 0.001<br>250 µM H_2O_2 + fucoidan vs. 250 µM H_2O_2:<br>1-50 µg/ml: *P* = 1.000
FV2	Z = 0.211, *P* < 0.001	F(9,70) = 3.359, *P* = 0.002	[chi]2(9,*N* = 80) = 62.416, *P* < 0.001, η^2 = 0.790	250 µM H_2O_2 vs. control: *P* = 0.002<br>250 µM H_2O_2 + fucoidan vs. 250 µM H_2O_2:<br>1-50 µg/ml: *P* = 0.992
FV3	Z = 0.229, *P* < 0.001	F(9,70) = 2.354, *P* = 0.022	[chi]2(9,*N* = 80) = 61.882, *P* < 0.001, η^2 = 0.783	250 µM H_2O_2 vs. control: *P* = 0.002<br>250 µM H_2O_2 + fucoidan vs. 250 µM H_2O_2:<br>1-50 µg/ml: *P* = 1.000

FS	Z = 0.226, $P < 0.001$	$F(9,60) = 2.072$, $P = 0.046$	[chi]2(9,N = 70) = 57.434, $P < 0.001$, $\eta^2 = 0.832$	250 μM H_2O_2 vs. control: $P = 0.004$<br>250 μM H_2O_2 + fucoidan vs. 250 μM H_2O_2:<br>1-50 μg/ml: $P = 1.000$
FE	Z = 0.232, $P < 0.001$	$F(9,50) = 3.764$, $P = 0.001$	[chi]2(9,N = 60) = 49.196, $P < 0.001$, $\eta^2 = 0.834$	250 μM H_2O_2 vs. control: $P = 0.010$<br>250 μM H_2O_2 + fucoidan vs. 250 μM H_2O_2:<br>1-5 μg/ml: $P = 1.000$, 10 μg/ml: $P = 0.801$, 50 μg/ml: $P = 1.000$
Fuc1	Z = 0.190, $P < 0.001$	$F(9,50) = 5.600$, $P < 0.001$	[chi]2(9,N = 60) = 45.491, $P < 0.001$, $\eta^2 = 0.771$	250 μM H_2O_2 vs. control: $P = 0.010$<br>250 μM H_2O_2 + fucoidan vs. 250 μM H_2O_2:<br>1-50 μg/ml: $P = 1.000$

Table S6. Detailed statistical analyses of HT-22 cells exposed to 0.35 μM erastin and fucoidan for 24 hours.

	Kolmogorov-Smirnov test	Levené test	Kruskal-Wallis test	Post hoc Mann-Whitney U with Bonferroni-Holm correction
FVs	Z = 0.205, *P* < 0.001	F(9,50) = 4.001, *P* = 0.001	[chi]2(9,*N* = 60) = 49.291, *P* < 0.001, η^2 = 0.835	0.35 μM erastin vs. control: *P* = 0.010<br>0.35 μM erastin + fucoidan vs. 0.35 μM erastin:<br>1-10 μg/ml: *P* = 0.016, 50 μg/ml: *P* = 0.200
FVm	Z = 0.201, *P* < 0.001	F(9,50) = 4.320, *P* < 0.001	[chi]2(9,*N* = 60) = 44.747, *P* < 0.001, η^2 = 0.809	0.35 μM erastin vs. control: *P* = 0.010<br>0.35 μM erastin + fucoidan vs. 0.35 μM erastin:<br>1-50 μg/ml: *P* = 1.000
UPm	Z = 0.246, *P* < 0.001	F(9,50) = 6.034, *P* < 0.001	[chi]2(9,*N* = 60) = 46.279, *P* < 0.001, η^2 = 0.784	0.35 μM erastin vs. control: *P* = 0.010<br>0.35 μM erastin + fucoidan vs. 0.35 μM erastin:<br>1-50 μg/ml: *P* = 1.000
FV1	Z = 0.213, *P* < 0.001	F(9,50) = 4.994, *P* < 0.001	[chi]2(9,*N* = 60) = 49.314, *P* < 0.001, η^2 = 0.836	0.35 μM erastin vs. control: *P* = 0.010<br>0.35 μM erastin + fucoidan vs. 0.35 μM erastin:<br>1 μg/ml: *P* = 0.525, 5 μg/ml: *P* = 0.149, 10 μg/ml: *P* = 0.749, 50 μg/ml: *P* = 0.149
FV2	Z = 0.200, *P* < 0.001	F(9,50) = 3.196, *P* = 0.004	[chi]2(9,*N* = 60) = 44.811, *P* < 0.001, η^2 = 0.760	0.35 μM erastin vs. control: *P* = 0.010<br>0.35 μM erastin + fucoidan vs. 0.35 μM erastin:<br>1-50 μg/ml: *P* = 1.000
FV3	Z = 0.195, *P* < 0.001	F(9,50) = 1.789, *P* = 0.094	[chi]2(9,*N* = 60) = 45.782, *P* < 0.001, η^2 = 0.776	0.35 μM erastin vs. control: *P* = 0.010<br>0.35 μM erastin + fucoidan vs. 0.35 μM erastin:<br>1-50 μg/ml: *P* = 1.000

FS	Z = 0.179, *P* < 0.001	F(9,50) = 2.917, *P* = 0.007	[chi]2(9,*N* = 60) = 44.854, *P* < 0.001, η^2 = 0.760	0.35 μM erastin vs. control: *P* = 0.010<br>0.35 μM erastin + fucoidan vs. 0.35 μM erastin:<br>1-50 μg/ml: *P* = 1.000
FE	Z = 0.172, *P* < 0.001	F(9,50) = 2.393, *P* = 0.024	[chi]2(9,*N* = 60) = 45.571, *P* < 0.001, η^2 = 0.772	0.35 μM erastin vs. control: *P* = 0.010<br>0.35 μM erastin + fucoidan vs. 0.35 μM erastin:<br>1-50 μg/ml: *P* = 1.000
Fuc1	Z = 0.178, *P* < 0.001	F(9,50) = 2.537, *P* = 0.018	[chi]2(9,*N* = 60) = 46.860, *P* < 0.001, η^2 = 0.794	0.35 μM erastin vs. control: *P* = 0.010<br>0.35 μM erastin + fucoidan vs. 0.35 μM erastin:<br>1-50 μg/ml: *P* = 1.000
Ferrostatin-1	Z = 0.310, *P* < 0.001	F(3,16) = 7.654, *P* = 0.002	[chi]2(3,*N* = 20) = 15.275, *P* = 0.002, η^2 = 0.804	0.35 μM erastin vs. control: *P* = 0.011<br>0.35 μM erastin + ferrostatin-1 vs. 0.35 μM erastin: *P* = 0.011

Table S7. Detailed statistical analyses of SH-SY5Y cells exposed to 30 μM erastin and fucoidan for 24 hours.

	Kolmogorov-Smirnov test	Levené test	Kruskal-Wallis test	Post hoc Mann-Whitney U with Bonferroni-Holm correction
FVs	Z = 0.260, *P* < 0.001	F(9,50) = 1.818, *P* = 0.088	[chi]2(9,*N* = 60) = 47.225, *P* < 0.001, η^2 = 0.800	30 μM erastin vs. control: *P* = 0.010<br>30 μM erastin + fucoidan vs. 30 μM erastin:<br>1-5 μg/ml: *P* = 0.313, 10 μg/ml: *P* = 0.631, 50 μg/ml: *P* = 0.458
FVm	Z = 0.180, *P* < 0.001	F(9,50) = 2.680, *P* = 0.013	[chi]2(9,*N* = 60) = 48.297, *P* < 0.001, η^2 = 0.819	30 μM erastin vs. control: *P* = 0.010<br>30 μM erastin + fucoidan vs. 30 μM erastin:<br>1-50 μg/ml: *P* = 0.598
UPm	Z = 0.216, *P* < 0.001	F(9,50) = 7.232, *P* < 0.001	[chi]2(9,*N* = 60) = 48.985, *P* < 0.001, η^2 = 0.830	30 μM erastin vs. control: *P* = 0.010<br>30 μM erastin + fucoidan vs. 30 μM erastin:<br>1 μg/ml: *P* = 0.601, 5-10 μg/ml: *P* = 0.673, 50 μg/ml: *P* = 0.313
FV1	Z = 0.255, *P* < 0.001	F(9,50) = 2.505, *P* = 0.019	[chi]2(9,*N* = 60) = 47.787, *P* < 0.001, η^2 = 0.810	30 μM erastin vs. control: *P* = 0.010<br>30 μM erastin + fucoidan vs. 30 μM erastin:<br>1 μg/ml: *P* = 0.078, 5-10 μg/ml: *P* = 0.075, 50 μg/ml: *P* = 0.065
FV2	Z = 0.208, *P* < 0.001	F(9,50) = 4.117, *P* = 0.001	[chi]2(9,*N* = 60) = 45.144, *P* < 0.001, η^2 = 0.765	30 μM erastin vs. control: *P* = 0.010<br>30 μM erastin + fucoidan vs. 30 μM erastin:<br>1-50 μg/ml: *P* = 1.000
FV3	Z = 0.237, *P* < 0.001	F(9,50) = 2.059, *P* = 0.052	[chi]2(9,*N* = 60) = 45.781, *P* < 0.001, η^2 = 0.776	30 μM erastin vs. control: *P* = 0.010<br>30 μM erastin + fucoidan vs. 30 μM erastin:<br>1-50 μg/ml: *P* = 1.000
FS	Z = 0.224, *P* < 0.001	F(9,50) = 3.730,	[chi]2(9,*N* = 60) = 46.224, *P* < 0.001,	30 μM erastin vs. control: *P* = 0.010

		P = 0.001	η^2 = 0.783	30 μM erastin + fucoidan vs. 30 μM erastin:<br>1-50 μg/ml: *P* = 1.000
FE	Z = 0.201, *P* < 0.001	F(9,50) = 3.075,<br>*P* = 0.005	[chi]2(9,*N* = 60) = 47.749, *P* < 0.001,<br>η^2 = 0.809	30 μM erastin vs. control: *P* = 0.010<br>30 μM erastin + fucoidan vs. 30 μM erastin:<br>1-50 μg/ml: *P* = 1.000
Fuc1	Z = 0.210, *P* < 0.001	F(9,50) = 3.218,<br>*P* = 0.004	[chi]2(9,*N* = 60) = 48.272, *P* < 0.001,<br>η^2 = 0.818	30 μM erastin vs. control: *P* = 0.010<br>30 μM erastin + fucoidan vs. 30 μM erastin:<br>1-10 μg/ml: *P* = 1.000, 50 μg/ml: *P* = 0.801
Ferrostatin-1	Z = 0.257, *P* = 0.001	F(3,16) = 3.492,<br>*P* = 0.040	[chi]2(3,*N* = 20) = 16.459, *P* = 0.001,<br>η^2 = 0.866	30 μM erastin vs. control: *P* = 0.011<br>30 μM erastin + ferrostatin-1 vs. 30 μM erastin: *P* = 0.465

Table S8. Detailed statistical analyses of primary cortical neurons exposed to 0.5 μM erastin and fucoidan for 24 hours.

	Kolmogorov-Smirnov test	Levené test	Kruskal-Wallis test	Post hoc Mann-Whitney U with Bonferroni-Holm correction
FVs	Z = 0.211, $P < 0.001$	$F(9,50) = 2.161$, $P = 0.041$	[chi]2(9,*N* = 60) = 45.233, $P < 0.001$, $\eta^2 = 0.767$	0.5 μM erastin vs. control: $P = 0.010$<br>0.5 μM erastin + fucoidan vs. 0.5 μM erastin:<br>0.1-5 μg/ml: $P = 0.437$
FVm	Z = 0.210, $P < 0.001$	$F(9,50) = 2.628$, $P = 0.014$	[chi]2(9,*N* = 60) = 46.032, $P < 0.001$, $\eta^2 = 0.780$	0.5 μM erastin vs. control: $P = 0.010$<br>0.5 μM erastin + fucoidan vs. 0.5 μM erastin:<br>0.1-5 μg/ml: $P = 1.000$
UPm	Z = 0.211, $P < 0.001$	$F(9,50) = 3.081$, $P = 0.005$	[chi]2(9,*N* = 60) = 46.690, $P < 0.001$, $\eta^2 = 0.791$	0.5 μM erastin vs. control: $P = 0.010$<br>0.5 μM erastin + fucoidan vs. 0.5 μM erastin:<br>0.1 μg/ml: $P = 0.749$, 0.5-1 μg/ml: $P = 0.449$, 5 μg/ml: $P = 0.313$
FV1	Z = 0.147, $P = 0.003$	$F(9,50) = 2.167$, $P = 0.041$	[chi]2(9,*N* = 60) = 45.190, $P < 0.001$, $\eta^2 = 0.766$	0.5 μM erastin vs. control: $P = 0.010$<br>0.5 μM erastin + fucoidan vs. 0.5 μM erastin:<br>0.1-5 μg/ml: $P = 1.000$
FV2	Z = 0.135, $P = 0.008$	$F(9,50) = 2.879$, $P = 0.008$	[chi]2(9,*N* = 60) = 44.990, $P < 0.001$, $\eta^2 = 0.763$	0.5 μM erastin vs. control: $P = 0.010$<br>0.5 μM erastin + fucoidan vs. 0.5 μM erastin:<br>0.1-5 μg/ml: $P = 1.000$
FV3	Z = 0.183, $P < 0.001$	$F(9,50) = 3.958$, $P = 0.001$	[chi]2(9,*N* = 60) = 46.217, $P < 0.001$, $\eta^2 = 0.783$	0.5 μM erastin vs. control: $P = 0.010$<br>0.5 μM erastin + fucoidan vs. 0.5 μM erastin:<br>0.1-5 μg/ml: $P = 1.000$
FS	Z = 0.161, $P = 0.001$	$F(9,50) = 1.698$,	[chi]2(9,*N* = 60) = 43.851, $P < 0.001$,	0.5 μM erastin vs. control: $P = 0.010$

		P = 0.114	η^2 = 0.743	0.5 μM erastin + fucoidan vs. 0.5 μM erastin:<br>0.1-5 μg/ml: P = 1.000
FE	Z = 0.186, P < 0.001	F(9,50) = 2.444,<br>P = 0.022	[chi]2(9,N = 60) = 46.595, P < 0.001,<br>η^2 = 0.790	0.5 μM erastin vs. control: P = 0.010<br>0.5 μM erastin + fucoidan vs. 0.5 μM erastin:<br>0.1-0.5 μg/ml: P = 0.112, 1 μg/ml: P = 0.065, 5 μg/ml: P = 0.423
Fuc1	Z = 0.200, P < 0.001	F(9,50) = 2.877,<br>P = 0.008	[chi]2(9,N = 60) = 48.351, P < 0.001,<br>η^2 = 0.820	0.5 μM erastin vs. control: P = 0.010<br>0.5 μM erastin + fucoidan vs. 0.5 μM erastin:<br>0.1-0.5 μg/ml: P = 0.847, 1 μg/ml: P = 0.328, 5 μg/ml: P = 0.149
Ferrostatin-1	Z = 0.212, P = 0.019	F(3,16) = 20.026,<br>P < 0.001	[chi]2(3,N = 20) = 13.489, P = 0.004,<br>η^2 = 0.710	0.5 μM erastin vs. control: P = 0.011<br>0.5 μM erastin + ferrostatin-1 vs. 0.5 μM erastin: P = 0.011
